# Oral probiotic activities and biosafety of *Lactobacillus gasseri* HHuMIN D

**DOI:** 10.1186/s12934-021-01563-w

**Published:** 2021-03-23

**Authors:** Soyon Mann, Myeong Soo Park, Tony V. Johnston, Geun Eog Ji, Keum Taek Hwang, Seockmo Ku

**Affiliations:** 1 Department of Food and Nutrition, and Research Institute of Human Ecology, Seoul National University, Seoul, 08826 Korea; 2 Research Center, BIFIDO Co., Ltd, Hongcheon, 25117 Korea; 3 Fermentation Science Program, School of Agriculture, College of Basic and Applied Sciences, Middle Tennessee State University, Murfreesboro, TN 37132 USA

**Keywords:** Antimicrobial Effect, *Lactobacillus gasseri*, Oral Microorganisms, Biosafety Evaluation

## Abstract

**Background:**

*Lactobacillus* spp. have been researched worldwide and are used in probiotics, but due to difficulties with laboratory cultivation of and experimentation on oral microorganisms, there are few reports of *Lactobacillus* spp. being isolated from the oral cavity and tested against oral pathogens. This research sought to isolate and determine the safety and inhibitory capabilities of a *Lactobacillus* culture taken from the human body*.*

**Results:**

One organism was isolated, named “*L. gasseri* HHuMIN D”, and evaluated for safety. A 5% dilution of *L. gasseri* HHuMIN D culture supernatant exhibited 88.8% inhibition against halitosis-producing anaerobic microorganisms and the organism itself exhibited powerful inhibitory effects on the growth of 11 oral bacteria. Hydrogen peroxide production reached 802 μmol/L after 12 h and gradually diminished until 24 h, it efficiently aggregated with *P. catoniae* and *S. sanguinis*, and it completely suppressed *S. mutans*-manufactured artificial dental plaque. *L. gasseri* HHuMIN D’s KB cell adhesion capacity was 4.41 cells per cell, and the cell adhesion of *F. nucleatum* and *S. mutans* diminished strongly in protection and displacement assays.

**Conclusion:**

These results suggest that *L. gasseri* HHuMIN D is a safe, bioactive, lactobacterial food ingredient, starter culture, and/or probiotic microorganism for human oral health.

## Introduction

Oral diseases are acute and chronic with a high prevalence globally, and oral health is a primary component of human health sustenance. Oral health is associated with a wide variety of illnesses and disorders, including dental caries, periodontal disease, tooth loss, oral cancer, oral symptoms of HIV infection, oral-dental trauma, and birth defects such as noma and cleft lip. Oral diseases cause suffering and disability to millions of Americans, costing taxpayers billions of dollars annually, according to the Centers for Disease Control and Prevention (CDC). According to a survey published in 2019, more than 40 percent of US adults report having felt pain in their mouths, and more than 80 percent have had at least one cavity by age 34. [1]. The 2015 Global Burden of Disease Survey estimated that about 3.5 billion people worldwide experience oral illnesses [2] and the World Health Organization (WHO) reports that millions of individuals suffer immensely from unhealthy oral cavities. Over the past 30 years, the global burden of tooth decay has remained largely unchanged and the total burden of oral disease will rise as a result of population growth and aging. Since the Tokyo Declaration on Dental Care and Oral Health for Healthy Lifetime, numerous oral health enhancement practices, including discussion of the overall value of oral health, the improvement of dental treatment systems, and the establishment of oral health programs, have been introduced by academia and industry [3].

Dental caries is caused by acid, formed by bacteria attached to the tooth's surface, which corrodes the enamel and dentine of teeth. *Streptococcus mutans* are Gram-positive, facultative anaerobic bacteria with a clear association with early dental caries in humans and animals. Insoluble glucan, combined by glucosyltransferase (GTase) in foods with sucrose and produced by *S. mutans*, has a high rate of adhesion to tooth and firm oral surfaces, and plays an important role in the development of dental caries. Oral microorganisms produce and attach glucans to oral surfaces, forming dental plaques and producing lactic acid (and others) during carbohydrate metabolism [4, 5].

Halitosis, defined as “stink through the mouth or nasal cavity” or “unpleasant odor from the mouth”, is caused by volatile sulfur compounds (VSCs) such as hydrogen sulfide (H_2_S) and methyl mercaptan (CH_3_SH) [6]. VSCs are produced by the decaying action of decomposing epithelial and white blood cells, which are eliminated by oral anaerobic bacteria. Volatile sulfur compounds are generated from sulfur-containing amino acids such as cysteine and methionine. Bacterial genera that produce hydrogen sulfide from cysteine include *Peptostreptococcus*, *Eubacterium*, *Selenomonas*, *Centipeda*, *Bacteroides*, *Fusobacterium,* and *Prevotella,* and genera that make methyl mercaptan from methionine are known to include *Fusobacterium*, *Bacteroides*, *Porphyromonas*, and *Eubacterium* [6, 7]. *F. nucleatum*, for example, a major hydrogen sulfide or methyl mercaptan producing, Gram-negative anaerobic bacterium, causes periodontitis and halitosis.

Many studies have been published on the inhibition of harmful oral bacteria [8–11]. Antibiotics such as penicillin, erythromycin, and tetracycline are effective in the prevention of oral disease, but they have not been utilized due to the overall increase in antibiotic resistance by bacteria [12]. Other studies have sought to inhibit oral periodontal bacteria using natural materials [11, 13, 14], inhibit GTase activity involving sucrose glucan formation [15], or use sucrose-alternative sweeteners that *S. mutans* is unable to metabolize [16]. Zinc ions, chlorhexidine (CHX) and cetylpyridinium chloride (CPC) and metal salts have been found to be effective in inhibiting the growth of halitosis-causing bacteria or in reducing the production of volatile sulfur compounds. Additionally, zinc chloride has been shown to mitigate halitosis by eliminating oral anaerobic bacteria [17]. Chlorhexidine, which is frequently used as a disinfectant in dental clinics, kills bacteria when applied to tooth enamel or the salivary pellicle and is widely used as a disinfectant in patients with periodontitis and to minimize halitosis [18]. Unfortunately, these strategies are not specific to harmful oral microorganisms or have a short period of action, making lasting effects difficult to achieve [19].

Microorganisms present in the oral cavity suppress the growth of fungi such as *Candida*, pathogenic microorganisms such as purulent bacteria, and viruses [20]. Therefore, a prevention strategy which suppresses the growth of all microorganisms present in the oral cavity is inappropriate for the prevention of oral disease. A healthy oral cavity can be maintained by promoting advantageous bacteria in the oral cavity and inhibiting bacteria that cause oral disease [21].

The Korean Ministry of Food and Drug Health requires manufacturers to submit information on the sources of microorganisms used in foods and drugs to allow for food product authorization and regulatory review for pharmaceutics. It has also developed a standard framework for quality assurance of these goods. The Korean National Institute of Food and Drug Safety Evaluation has also laid out standard guidelines for the quality assurance of probiotics products [23]. An estimation of the safety level of isolated bacteria for novel clinical probiotic use is important for the identification and elimination of possible clinical pathogens.

In this study, *Lactobacillus* spp. were evaluated as a means of controlling harmful oral microorganisms. *Lactobacillus* bacteria that inhibit the growth of harmful oral bacteria were collected from the saliva and feces of adults over 20 years of age. One individual species was isolated and identified, and safety tests were carried out following Food and Agriculture Organization of the United Nations (FAO)/World Health Organization (WHO)-recommended studies and other published safety tests to verify its safety for academic and commercial applications [24].

## Materials and methods

### Collection and isolation of lactic acid bacteria

Samples of saliva were taken from 200 adults over 20 years of age with little or no supragingival plaque, enrolled in Seoul National University's Food Microbiological Research Laboratory (IRB No. 1907/003-018). Before brushing and before meals, samples were taken in the morning, combined with sterile 70 percent glycerol (1:1), and refrigerated until tested. Fecal samples were taken from 28 people over 20 years of age recruited from the Catholic University of Korea, Seoul St. Mary’s Hospital (IRB KC17TNSI0570). Feces was collected in stool containers and stored frozen until analyzed. *Lactobacillus* colonies with excellent hydrogen peroxide production were selected using the process defined by Eschenbach et al. [25] and John F. T. Spencer [26]. In keeping with their methods, two solutions, A and B, were prepared to add tetra methyl benzidine (3.3′, 5.5′-tetra methyl benzidine or TMB) agar as a screening agent. Solution A consisted of 3 mL of DMSO (dimethyl sulfoxide, Sigma, St Louis, MO, USA) and 12.5 mg of TMB (Sigma, St Louis, MO). Solution B (0.5 mg/mL or 100 U/mL peroxidase) consisted of 1 mL of sterilized water and 0.5 mg of peroxidase (Sigma, St Louis, MO, USA). de Man-Rogosa-Sharpe (MRS, BD Difco™, Franklin Lakes, NJ, USA) agar was sterilized at 121 ℃ for 15 min and brought to 45 ℃. To create the final selective agar medium, 0.6 mL of Solution A and 0.2 mL of Solution B per 10 mL of MRS agar was added. The mixture was poured and allowed to solidify on plates. Each selection plate, therefore, had a concentration of 1 mM TMB and 10 μg/mL (2 U/mL) of peroxidase.

To isolate lactic acid bacteria producing hydrogen peroxide, 100 μL or μg of each obtained sample was diluted serially with sterilized phosphate buffered saline (PBS, pH 7.4). The diluted solution was then distributed across the TMB selective agar plates. After 48 h of anaerobic incubation at 37 °C, the samples were exposed to 15–30 min of oxygen to allow for indicator response. Horseradish peroxidase induces oxidation of TMB by bacteria via the production of hydrogen peroxide, forming a blue colony. If the colony was blue, it was assessed as producing hydrogen peroxide ( +). Ten single colonies of blue color formed on each medium were randomly chosen for multiple streaking onto new TMB agar plates to separate 500 single colonies.

### Challenge and control bacterial strains and growth conditions

Four oral anaerobic bacteria (*Fusobacterium nucleatum* KCOM 1001, *Porphyromonas gingivalis* KCOM 2796, *Prevotella intermedia* KCOM 2889, and *Porphyromonas catoniae* KCOM3169) and seven oral facultative anaerobic bacteria (*Streptococcus sobrinus* KCOM1157, *Streptococcus mitis* KCOM 1356, *Streptococcus oralis* KCOM 1493, *Streptococcus gordonii* KCOM 1788, *Streptococcus sanguinis* KCOM 2167, *Streptococcus parasanguinis* KCOM 2522, and *Streptococcus mutans* KCTC3065) from the Korean Collections of Oral Microbiology were utilized as challenge strains. *F. nucleatum, P. gingivalis*, *P. intermedia*, and *P. catoniae* KCOM3169 were individually incubated in KCOM broth (Brain Heart Infusion (BHI, BD Difco™, Franklin Lakes, NJ, USA) with 0.5% yeast extract (BD Difco™, Franklin Lakes, NJ, USA), 0.05% Cysteine HCl-H_2_O (Sigma, St Louis, MO, USA), 0.025% Resazurin (Sigma, St Louis, MO, USA), 5 mg/mL Hemin solution (Sigma, St Louis, MO, USA), and 10 mg/mL vitamin K1 solution (Sigma, St Louis, MO, USA) for 24 h at 37 °C under anaerobic conditions (85% N_2_, 10% H_2_, 5% CO_2_). *S. sobrinus* KCOM1157, *S. mitis* KCOM 1356, *S. oralis* KCOM 1493, *S. gordonii* KCOM 1788, *S. sanguinis* KCOM 2167, *S. parasanguinis* KCOM 2522, and *S. mutans* KCTC3065 were individually grown in BHI broth under aerobic conditions for 24 h at 37 ℃ before use. Prior to use in each experiment, strains were subcultured twice in the appropriate medium. *Weissella* cibaria, a commercially available oral probiotic used as a *Lactobacillus* control in this study, was inoculated into MRS broth and incubated at 37° C for 24 h before use.

### Evaluation of the antibacterial effect of isolated lactic acid bacteria

Fifty hydrogen peroxide producing *Lactobacillus* isolates were individually incubated at 37 °C in 5 ml of traditional MRS broth for 24 h, and their supernatants were recovered by centrifugation at 4 °C for 5 min at 25,000 × g. To remove the antimicrobial influence of organic acids, the supernatants were adjusted to pH 6.5 ~ 7 with 1 M syringe sterilized (0.45 μm) NaOH and stored at − 20 °C before analysis. Two hundred μL of MRS broth was added to 96-well plates (SPL Life Sciences Co., Ltd. Pocheon, Korea) and the 11 oral microorganisms were inoculated at 1% into each well. Ten μL of each hydrogen peroxide producing *Lactobacillus* isolate supernatant was added to the wells seeded with oral microorganisms (final concentration 5%) and the plates were anaerobically incubated at 37 °C. The wells were read at 600 nm for up to 24 h using a microplate reader (Epoch2, BioTek, Winooski, VT, USA) to determine the level of inhibition by the supernantant. One *Lactobacillus* isolate was designated as "HHuMIN D" based on its superior inhibition of oral microorganism growth. 16S rRNA sequencing was conducted to genetically identify the isolated bacteria, followed by base-sequence molecular phylogenetic analysis. The organism was identified as *L. gasseri* and named "*L. gasseri* HHuMIN D". For further experimental use, *L. gasseri* HHuMIN D was inoculated into MRS broth and incubated at 37° C for 24 h. The culture broth of *L. gasseri* HHuMIN D was placed in 50% (v/v) glycerol solution and stored at − 80 °C.

### Evaluation of the effect of *L. *gasseri HHuMIN D on oral microorganisms

To test the impact of *L. gasseri* HHuMIN D on oral microorganisms, viable cell counts were carried out after co-culture using selective agar plates. The 11 challenge oral bacteria and *L. gasseri* HHuMIN D were inoculated at 5% in a 50% KCOM broth/50% BHI broth medium in test tubes. The tubes were incubated at 37 °C for 24 h, followed by viable cell counts for each bacterium. All tubes were serially diluted after incubation; anaerobes were plated on KCOM agar, facultative anaerobes and anaerobes were plated on MSB agar (Mitis Salivarius Sucrose Bacitracin, Sigma-Aldrich, St. Louis, MO, USA), and *L. gasseri* HHuMIN D was plated on LBS agar (Sigma-Aldrich, St. Louis, MO, USA). This experiment was repeated three times, and the average value was determined after calculating the inhibition rate for each replication as % inhibition = 100 × (MAX—MIN) / MAX (where MAX = viable cell count with no inhibition and MIN = viable cell count after co-culture).

### Hydrogen peroxide production test

*L. gasseri* HHuMIN D culture medium, prepared as previously described, was centrifuged for 10 min at 4° C at 2000 × g, and the supernatant extracted. Supernatants were colorimetrically analyzed using Pierce Quantitative Peroxide Assay Kits (aqueous-compatible formulation, Thermo Fisher Scientific, Waltham, MA, USA). Two hundred μL of working reagent (containing ammonium ferrous (II) sulfate, sorbitol, xylenol orange) and 20 μL of *L. gasseri* HHuMIN D supernatant were placed into individual cells of a 96-well microplate and allowed to develop for fifteen minutes. A 1 mM (1000 µM) solution of hydrogen peroxide was produced to standardize hydrogen peroxide production by diluting a 30% H_2_O_2_ supply 1:9000 (11µL of 30% H_2_O_2_ into 100 mL of ultrapure water). The 1 mM hydrogen peroxide solution was then serially diluted 1:2 with sterile water (100 μL of sterile water + 100 μL of 1 mM hydrogen peroxide). Two hundred μL of the working reagent was then added to 20 μL of the diluted H_2_O_2_ standard in microplate wells. The absorbance of both the *L. gasseri* HHuMIN D and hydrogen peroxide standard plate wells were assessed via microplate reader at 595 nm (Epoch2, BioTek, Winooski, VT, USA).

### Susceptibility assay of the bacteriocin produced by *L. gasserie* HHuMIN D to hydrolytic enzymes

Proteins, carbohydrates and lipolytic enzymes were purchased from Sigma Aldrich (St. Louis, MO, USA) and used according to the manufacturer's instructions. Lipase (4307 U/mg) and trypsin (13,50 U/mg) in 50 mM Tris–HCl buffer (pH 7.5), α-chymotrypsin (83.9 U/mg) and carboxypeptidase A (73 U/mg) in 50 mM Tris–HCl (pH 8.0), pepsin (3.280 U/mg) in 10 mM citrate (pH 6.0), α-amylase (519 U/mg) in 0.1 M sodium phosphate (pH 7.0) and proteinase K (30 U) /mg) in 0.01 M Tris–HCl (pH 7.9), 0.05 M EDTA and 0.5% SDS buffer were prepared at a concentration of 20 mg/mL. The enzymes were added to the culture supernatant at 10% (2 mg/mL) and reacted at 37 ℃ for 150 min except for proteinase K, which was reacted at 45 ℃ for 12 h. The remaining bacteriocin activity was measured using a microtiter plate for oral harmful bacteria. A sample not treated with an enzyme solution under the same conditions was used as a negative control [27].

### Coaggregation assays

*L. gasseri* HHuMIN D coaggregation was tested using the challenge microorganisms and a modified Handley et al. [28] spectrophotometric assay. *L. gasseri* HHuMIN D cells were harvested by centrifuging the culture medium at 4000 × g for 15 min. Pellets were washed with Cisar's buffer three times and resuspended to approximately 10^9^ cells/mL. One ml of the coaggregation pairs were vortexed for 10 s and incubated with soft stirring at 110 rpm at 37 °C for 30 min. The coaggregations were then allowed to remain at room temperature for 3 min after incubation. One half ml of supernatant was removed from the culture and the optical density at 660 nm was determined using a microplate reader (Epoch2, BioTek, Winooski, VT, USA).

Coaggregation was determined using the decrease in absorbance vs. the control. The proportion of coaggregation was determined using the equation by Handley et al. (1987):

$${\text{Coaggregation }}\left( \% \right) \, = \frac{{{\text{OD}}_{{{66}0}} \left( {{\text{X control }} + {\text{ Y control}}} \right)/{2} - {\text{OD}}_{{{66}0}} \left( {{\text{X }} + {\text{ Y}}} \right) \times {1}00 }}{{ {\text{OD}}_{{{66}0}} \left( {{\text{X control }} + {\text{ Y control}}} \right)/{2}}}$$

where X control and Y control represent OD_660_ of the two microorganisms in the control tubes and (X + Y) is the mixture of organisms.

### Effect of *L. gasseri *HHuMIN D on the formation of artificial dental plaque by *S. mutans*

To assess whether and the degree to which *L. gasseri* HHuMIN D prevents the development of artificial dental plaque by *S. mutans*, beaker wire tests following the protocol of Yu et al. [29] were conducted. Five percent sucrose was added to 40 ml of broth consisting of equivalent quantities of BHI and MRS broth and the pH was adjusted to 7.0 by the addition of 0.1 M MOPS buffer. The medium was inoculated with 1 × 10^8^ *S. mutans* and 1 × 10^8^ *L. gasseri* HHuMIN D cells alone or combination. Stainless steel orthodontic wires (4 cm length, 0.016-inch, 1 mm dia, Oramco, Glendora, CA, USA) were suspended on conical tubing and immersed in the broth. The weights of the artificial dental plaques produced on the wires were calculated after 12 h of incubation at 37° C while shaking at 30 rpm.

### Adhesion assay of *L. gasseri* HHuMIN D

KB cell line oral epithelial cells (KCLB 10,017) were acquired from the Cell Line Bank of Korea (Seoul, Korea). KB cells were cultured in RPMI-1640 Media (Sigma-Aldrich, St. Louis, MO, USA) containing 10% (v/v) fetal bovine serum (FBS, Sigma-Aldrich, St. Louis, MO, USA) and 1% (v/v) antibiotic–antimycotic solution (Sigma-Aldrich, St. Louis, MO, USA) in 5% CO_2_ at 37 ℃. For the adhesion efficiency assay, KB cells were inoculated into a minimum critical medium (MEM, Sigma-Aldrich, St. Louis, MO, USA) containing 10 percent (v/v) FBS at 110^5^ cells/well. Upon transfer to a 24-well multi-culture dish (Thermo Fisher Scientific, St Peters, MO, USA), the cells were grown for 18 h at 5% CO_2_ at 37 °C.

*L. gasseri* HHuMIN D was grown for 18 h in MRS broth at 37 °C and the bacterial concentration was adjusted to 10^8^ CFU/mL (measured at 600 nm). Isolated bacteria were centrifuged at 8000 × g for 3 min and washed with PBS three times. The washed bacteria were then resuspended in MEM media. The number of cells measured by hemocytometer (Paul Marienfeld, Lauda-Königshofen, Germany) was about 6 log cells per well.

*L. gasseri* HHuMIN D cells were then added to the 24-well culture dish cells containing KB cells at about 6 log cells per well and incubated for 1 h at 37 °C and 5% CO_2_. The suspension was eliminated after incubation and the 24-well plates were washed two times with PBS (pH 7.4). For 10 min, 200 μL trypsin/EDTA (Welgene, Daegu, Korea) was applied to the cell monolayer, and the cells and attached bacteria were collected. MG cell Genomic DNA Extraction SV kits (Doctor Protein, Seoul, Korea) were used to remove the genomic DNA (gDNA) of the attached bacteria in the extracted pellets. Pending analysis, the final volume of all DNA samples (200 μL) was stored at -20 °C. The number of bacteria adhered to the KB cells was determined using qRT-PCR.

Protection and displacement assays were conducted to assess the competition between *L. gasseri* HHuMIN D and harmful oral bacteria (*F. nucleatum* and *S. mutans*) for cell adhesion using modified versions of a previously reported process [30]. In protection assays, 1 mL (110^8^ CFU/mL) of *L. gasseri* HHuMIN D was applied to 24-well culture dishes loaded with KB cells and incubated for 1 h at 37 °C and 5% CO_2_ for 1 h. The suspension was removed after incubation, and the cell monolayer was washed twice with PBS. Next, 1 mL (110^8^ CFU/mL) of a harmful oral bacteria suspension (*F. nucleatum* and *S. mutans*) was applied to the wells and incubated for 1 h at 37 °C and 5% CO_2_. The suspension was removed, and the cell monolayer was washed twice with PBS. Two hundred μL of trypsin/EDTA (Welgene, Daegu, Korea) was added to the KB cell monolayer and allowed to stand for 10 min, after which the KB cells and attached bacteria were gathered.

In displacement assays, 1 mL (110^8^ CFU/mL) of a harmful oral bacteria suspension (*F. nucleatum* and *S. mutans*) was applied to 24-well KB cells and incubated for 1 h at 37 °C and 5% CO_2_ for 1 h. The suspension was removed after incubation and the monolayer cell was washed twice with PBS. Next, 1 mL (110^8^ CFU/mL) of *L. gasseri* HHuMIN D was applied to the wells and incubated for 1 h at 37 °C and 5% CO_2_. After the second inoculation, the experimental procedure was the same as for the protection assays.

KB cells attached only to *F. nucleatum* and *S. mutans* were used as controls. The number of bacteria appended to each KB cell was determined using real-time PCR.

The collected gDNA was used to quantitatively analyze each bacterium that was attached to the KB cells using real-time PCR. Standard curves were drawn using bacterium-specific primers, and the bacteria were quantified to test cell adhesion from the extracted DNA. A standard curve was drawn using primers specific to each bacterium, and bacteria were quantified from the extracted DNA to measure cell adhesion. The primers used to amplify the bacteria are shown in Table 1. Real-time PCR was conducted on 96-well plates (SPL Life Sciences Co., Ltd. Pocheon, Korea) using tepOnePlus™ Real Time PCR systems (Thermo Fisher Science, St Peters, MO, USA). The PCR reaction was performed using 1 × TB Green™ Premix Ex Taq™ (Takara, Japan), and real-time PCR conditions are as follows; the first denaturation for 10 min at 95 °C, and then 40 polymerase chain reaction cycles at 95 °C for 15 s and 60 °C for 1 min. Analysis of the results was carried out using StepOne ™ and StepOnePlus ™ Software v2.3 (Thermo Fisher Scientific, St Peters, MO, USA). The number of bacteria was calculated by converting the value of the cycle threshold (CT) into the standard curve.

**Table 1:** Oligonucleotides used as primers for real-time PCR

Bacteria name	Oligonucleotide sequence		Length	Reference
*Lactobacillus*	Forward	TGG AAA CAG RTG CTA ATA CCG	21	[102]
	Reverse	GTC CAT TGT GGA AGA TTC CC	20	
*Fusobacterium nucleatum*	Forward	AGA GTT TGA TCC TGG CTC AG	20	[103]
	Reverse	GTC ATC GTG CAC ACA GAA TTG CTG	24	
*Streptococcus mutans*	Forward	CTA CAC TTT CGG GTG GCT TG	20	[104]
	Reverse	GAA GCT TTT CAC CAT TAG AAG CTG	24	

### Safety assessment of *L. gasseri* HHuMIN D

#### Ammonia production testing

The catalyzed indophenol reaction [31] ammonia production test was utilized. *L. gasseri* HHuMIN D cells were cultured anaerobically in BHI media at 37 °C for 5 days, after which the culture medium was centrifuged (2236R centrifuge; Labogene Aps, Lillerød, Denmark) at 10,000 × g for 30 min to isolate the supernatant. The supernatant was adjusted after extraction to pH 7.0.

Two solutions were prepared: Solution A consisted of 0.01 g of sodium nitroferricyanide dihydrate (Sigma, St. Louis, MO, USA) and 2 g of phenol (Sigma, St. Louis, MO, USA) in 200 mL LC–MS grade water, and Solution B consisted of 1 g of sodium hydroxide and 0.08 g of sodium hypochlorite in 200 mL LC–MS grade water. One hundred μL of *L. gasseri* HHuMIN D supernatant was dispensed into 96-well plates, followed by 10 μL each of solutions A and B, and incubated for 1 h at 20 °C. Using a microplate reader (Bio-Rad Laboratories, Philadelphia, PA, USA) the optical density was measured at 625 nm. Uninoculated BHI medium was used as a control and a standard curve was used to measure ammonia concentration. *Enterococcus faecium* ATCC19433 supernatant was employed as a positive control.

#### Biogenic amine production testing

Biogenic amine analysis was conducted using the method of Kim et al. [32]. Four biogenic amines, cadaverine (≧ 97.0%), histamine (≧ 97.0%), putrescine (≧ 98.5%), and tyramine (≧ 99.0%), were purchased from Sigma-Aldrich (St. Louis, MO, USA), as were 1.7-diaminoheptane (internal standard; ISTD, 98%, Cat. # D174708), dansyl chloride (≥ 99.0%, Cat. # 39,220), and L-proline (≥ 99.0%, Cat. # P0380). Whatman No. 4 filter paper was purchased from Whatman Intl., Ltd (Maidstone, UK). Sodium carbonate (99.0%, Cat. # 433,401,201), ether (99.0%, Cat. # 33475S1280), and acetone (99.7%, Cat. # A0108) were purchased from Samchun Pure Chemical Co., Ltd. (Pyeongtaek, Korea).

*L. gasseri* HHuMIN D was grown anaerobically in MRS broth with 0.05 percent (w/w) L-cysteine-HCl (Sigma, St. Louis, MO, USA) at 37 ℃ for 18 h. Next, 25 mL 0.1 N HCl was added to 5 mL of *L. gasseri* HHuMIN D supernatant and blended for 5 min. The supernatant was separated by centrifugation (2236 R centrifuge; Labogene Aps, Lillerød, Denmark) at 10,000 × g for 15 min. The gathered supernatant was filtered through a 20–25 μm membrane filter (Whatman Int'l., Ltd., Maidstone, UK) and 1 mL of filtrate was combined with 0.1 mL of 0.01% (w/v) 1.7-diaminoheptane, 0.5 mL of saturated sodium carbonate solution, and 1 mL of 1% dansyl chloride. The mixture was incubated in a water bath (WBC 1510A; Jeio Tech. Co., Ltd., Seoul, Korea) after thorough mixing, with light blocked at 45 ℃ for 1 h. By adding 0.5 mL of 10 percent proline and 5 mL diethyl ether at room temperature and allowing it to stand for 5 min, the remaining dansyl chloride was removed. The supernatant was concentrated using a Scanvac Speed Vacuum Concentrator (Labogene Aps, Lillerød, Denmark) at 1500 rpm and 20° C until dry for high performance liquid chromatography (HPLC) analysis. Next, the dry pellets were resuspended in 1 mL of acetonitrile (Sigma-Aldrich, St. Louis, MO, US). After filtering through a 0.22-μm filter membrane, the resuspended sample and standard were analyzed. A Thermo Dionex Ultimate 3000 HPLC (Thermo Fisher Scientific, St Peters, MO, USA) with a VDSpher C-18 column (4.6 250 mm, 5 µm) (VDS Optilab Chromatographie Technik GmbH, Berlin, Germany) was used to analyze the samples. The mobile phase was (A) acetonitrile, (B) distilled water (0–1 min, A: B = 40: 60; 1–25 min, A: B = 100: 0; 25—35 min, A: B = 60: 40). The injection volume was 20 μL, the flow rate was 0.8 mL/min, the column temperature was 30 ℃ (constant), and the UV detection wavelength was 250 nm.

#### Hemolysis testing

Hemolysis testing was conducted according to the method of Kim et al. [32]. Hemolysis was observed by anaerobic incubation of *L. gasseri* HHuMIN D in BL agar (BD Difco™, Franklin Lakes, NJ, USA) for 2 days at 37 °C with 5% sheep blood. As a positive control, *Listeria ivanovii* subsp. ivanovii ATCC 19,119 was incubated on the same agar for 2 days under aerobic conditions at 37 °C. The presence or absence of light visible through both sides of the plates indicated hemolysis. Strains that did not produce green areas around the colonies (α-hemolysis) or did not cause hemolysis in the blood plate (γ-hemolysis) were considered nonhemolytic. Strains having a clear zone around the colonies were classified as β-hemolytic bacteria.

#### Mucin degradation testing

The degradation of mucins was estimated using the method of Kim et al. [32]. Type III porcine stomach mucin was purchased from Sigma-Aldrich (St. Louis, MO, USA). As a negative control, MRS medium was used without a carbon source. Four MRS broths with different carbon sources were prepared: 0.5% (w/v) mucin, 1.0% (w/v) mucin, 0.5% (w/v) glucose, and 1% (w/v) glucose. Each broth was inoculated with *L. gasseri* HHuMIN D and anaerobically incubated 2 days at 37 °C. Strain growth was estimated using a microplate reader (Bio-Rad Laboratories, Philadelphia, PA, USA) at 0, 12, 24, 36, and 48 h by measuring the optical density at 550 nm. By subtracting the final optical density value of each sample from the original optical density value, the growth in each broth was determined.

#### Antimicrobial susceptibility testing

Antibiotic resistance may be present in *Lactobacillus* spp. [33] and since antibiotic resistance can be transmitted via plasmids, the evaluation of antibiotic resistance is an important consideration for their safety assessment [34]. *L. gasseri* HHuMIN D antimicrobial susceptibility was estimated using the method of Kim et al. [32]. Twenty-three antimicrobial compounds were tested in this experiment: ampicillin sodium salt, bacitracin, carbenicillin disodium salt, cephalothin sodium salt, chloramphenicol, clindamycin hydrochloride, dicloxacillin sodium salt hydrate, erythromycin, gentamicin sulfate, kanamycin sulfate, lincomycin, methicillin, metronidazole, mupirocin, neomycin sulfate, penicillin G, phosphomycin disodium salt, polymyxin B sulfate salt, rifampicin, streptomycin sulfate salt, tetracycline, trimethoprim–sulfamethoxazole (trimethoprim), and vancomycin hydrochloride.

All antimicrobials were purchased from Sigma (St. Louis, MO, USA) except vancomycin hydrochloride, which was purchased from USP, Bethesda, Maryland, USA. Before being added to LSM-Cys broth (LAB susceptibility test medium with L-cysteine, consisting of 90 percent IST and 10 percent MRS broth medium), each antibiotic powder was dissolved in a suitable diluent, sterilized and filtered. IST was purchased from Kisan Bio Co., Ltd. (Mb cell I so-Sensitest Broth, Seoul, Korea), and MRS was purchased from Becton, Dickinson and Company (BD Difco™ MRS *Lactobacilli* broth, Franklin Lakes, NJ, USA). The ISO10932: 2010 minimal inhibitory concentration (MIC) values ​​for the 20 antibiotics tested on *L. gasseri* HHuMIN D were used.

1. For this experiment, LSM-Cys (LAB susceptibility test) medium containing 0.03% (w/v) L-cysteine HCl was used with 96-well plates. Upon diluting serially twice, the antibiotic broth used ranged from 0.0032—1024 g/mL. Finally, the microorganisms were inoculated at a rate of 0.2% (the final concentration was approximately 310^8^ cfu/mL) in the LSM-Cys media and 50 μL of each diluted medium was inoculated into each well. There was no negative control. The dilute medium was anaerobically incubated at 37 °C for 48 h. The MIC was visually determined by comparing the test samples to the control; antibiotic susceptibility was considered to be the prevention of bacterial growth by 80% or more.

### Complete genome sequencing, assembly, and annotation of *L. gasseri* HHuMIN D

Chunlab, Inc. (Seoul, Korea), which used PacBio Sequel Systems (Pacific Biosciences, Menlo Park, CA, USA) and CLgenomicsTM and EZBioCloud Apps services (Chunlab, Seoul, Korea), conducted whole genome sequencing of *L. gasseri* HHuMIN D. General gene information was investigated by EZBiodCloud Apps and functional annotations for the expected genes were analyzed using RefSeq protein (NR; NCBI), Clusters of Orthologous Groups of proteins (COG), EggNOG, SEED, Swiss-Prot, and Kyoto Encyclopedia of Genes and Genomes (KEGG) databases [35–38].

### Safety evaluation with complete genome sequence

Analysis of virulence factors was carried out through the VirulenceFinder 2.0 Website, which is a part of the openly accessible web-based tool for whole genome sequencing (WGS) analysis hosted by the Center for Genomic Epidemiology (CGE, http://www.genomicepidemiology.org/, 2014). The predicted *L. gasseri* HHuMIN D genes were compared with the comprehensive antibiotic resistance database (CARD, http://arpcard.mcmaster.ca) for identifying antibiotic resistance (https://card.mcmaster.ca). The ability to synthesize biogenic amines and aggregate platelets was searched using full genome sequencing of *L. gasseri* HHuMIN D via CLGenomics (ChunLab, Seoul, South Korea, 2004) through matching with the KEGG pathway.

### Statistical analysis

Data are expressed as mean ± SEM by SPSS software (IBM SPSS Statistics, Version 25, SPSS Inc., Chicago, IL, USA). Differences between the means were measured by one-way ANOVA using Duncan’s multiple range test. Statistical significance was assumed at *p* < 0.05.

## Results and discussion

### Identification of hydrogen peroxide-generating *lactobacilli*

The screening of *Lactobacillus* progressed in a three-step process. First, 500 strains with excellent development of hydrogen peroxide were isolated from saliva and feces. Second, *Lactobacillus* sp. were collected using LBS agar plates to sort through the 500 isolated strains. A total of 50 isolates were identified. Finally, the 50 isolates were tested for their strength against oral microorganisms, and HHuMIN D was the most effective.

The antimicrobial effect of 5% of HHuMIN D supernatant was clearly demonstrated in the control of anaerobic oral bacteria causing halitosis. *P. catoniae, P. intermedia, F. nucleatum,* and *P. gingivalis were* inhibited by 90, 89 ± 1, 88 ± 1, and 88 ± 1%, respectively. *S. mutans, a* dental caries-inducing bacterium, was inhibited by 60 ± 2%. *S. mitis* and *S. gordonii,* which cause periodontitis, were inhibited by 22 ± 2 and 19 ± 5%, respectively. Antimicrobial activity was lower against oral facultative anaerobic bacteria (*S. sobrinus, S. sanguinis, S. parasanguinis*, and *S. oralis*). On the other hand, the antimicrobial effect of 5% of *W. cibaria* culture supernatant showed relatively weaker antimicrobial activity than HHuMIN D (Table 2).

**Table 2:** Inhibition of periodontal bacteria by *L. gasseri* HHuMIN D supernatant

Strains	Inhibition (%)	
	*W. cibaria*	*L. gasseri* HHuMIN D
*Fusobacterium nucleatum* KCOM 1001	5 ± 1^a^	**88 ± 1**^a^
*Porphyromonas gingivalis* KCOM 2796	11 ± 3^a^	**88 ± 1**^a^
*Prevotella intermedia* KCOM 2889	13 ± 1^a^	**89 ± 1**^a^
*Porphyromonas catoniae* KCOM3169	14 ± 2^a^	**90 ± 0**^a^
*Streptococcus sobrinus* KCOM1157	8 ± 1^a^	**60 ± 2**^b^
*Streptococcus mitis* KCOM 1356	17 ± 2^b^	4 ± 4^c^
*Streptococcus oralis* KCOM 1493	22 ± 5^b^	4 ± 6^c^
*Streptococcus gordonii* KCOM 1788	0 ± 0^c^	19 ± 5^d^
*Streptococcus sanguinis* KCOM 2167	0 ± 0^c^	2 ± 3^c^
*Streptococcus parasanguinis* KCOM 2522	12 ± 3^a^	2 ± 2^c^
*Streptococcus mutans* KCTC3065	0 ± 0^c^	22 ± 2^d^

Values are expressed as the mean ± standard deviation. Means not sharing a common letter are significantly different groups at *p* < 0.001 (n = 3)

*Lactobacillus* culture medium neutralization and disinfection is an experimental process used to monitor the antibacterial activity of bacteria metabolites, such as bacteriocin. In another study, sterile neutral *S. mutans* ATCC 25,175 supernatant showed > 80% inhibition of *L. paracasei* strains [39]. Another study confirmed that *Lactobacillus gasseri*, which produces gassericin A, has inhibitory properties against a wide range of oral pathogens, including carcinogenic and periodontal pathogens [40].

**Fig. 1:**
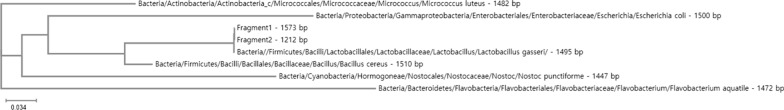
Most likely phylogenetic tree derived from the 16S rRNA sequence of *L. gasseri* HHuMIN D

HHuMIN D was shown to produce hydrogen peroxide and suppress oral microorganisms. 16S rRNA gene sequencing of HHuMIN D showed that it is a *Lactobacillus* sp. Multiple alignment of 16S rRNA base sequences of isolated bacteria with the *Lactobacillus* sp. 16S rRNA sequence was obtained through the similarity matrix listed on GenBank. This genealogy research showed a 99% 16S rRNA homology; HHuMIN D shares 99% homology with *L. gasseri* (Fig. 1).

*Lactobacillus* species isolated from the oral cavity vary widely. Lactic acid bacteria such as *L. acidophilus, L. casei, L. fermentum, L. gasseri, L. johnsonii, L. rhamnosus, L. reuteri, L. salivarius* and *L. vaginalis* are probiotic strains commonly used in dairy and health/functional food products [41–44]. The current research confirms that candidate probiotic *Lactobacillus* strains are present in the oral cavity and the probiotic capabilities of *L. gasseri* HHuMIN D.

### Inhibition effect of *L. gasseri* HHuMIN D on oral microorganisms

Oral lactic acid bacteria are known to inhibit harmful oral bacteria by the production of various antibiotics [45]. *L. gasseri* has been studied as an oral probiotic and results have shown that it is effective against harmful oral bacteria [46, 47].

**Fig. 2:**
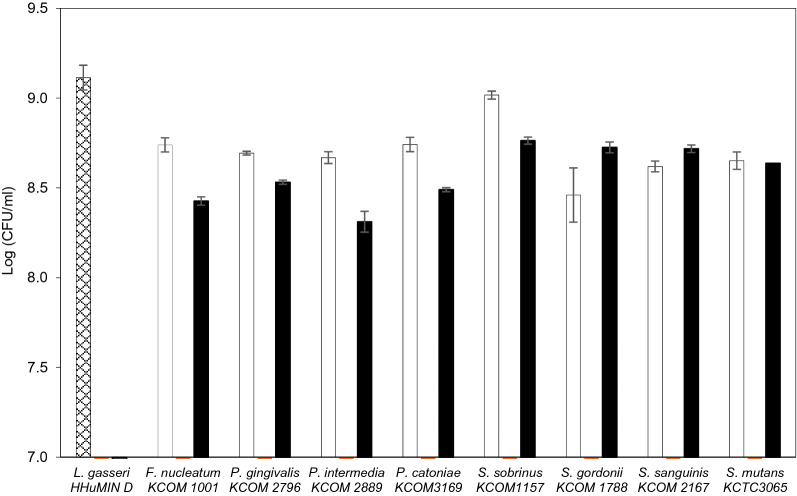
Inhibitory effect of *L. gasseri* HHuMIN D on the proliferation of various periodontal bacteria. ▩, *L. gasseri* HHuMIN D; □, Periodontal bacteria group; ▨, Periodontal bacteria in mixed culture; ■, *L. gasseri* HHuMIN D in mixed culture. Log 7 and below means < 10^5^

The interactions between *L. gasseri* HHuMIN D and oral anaerobic and facultative anaerobic bacteria are shown in Fig. 2. All harmful oral bacteria were strongly inhibited by *L. gasseri* HHuMIN D, but interaction with oral microorganisms also impaired its proliferation. Compared to single culture, *L. gasseri* HHuMIN D showed poor growth in most co-cultures. Among them, the lowest viable cell counts occurred when *L. gasseri* HHuMIN D was co-cultured with *P. intermedia*.

A previous study on the co-culture of oral anaerobia (*F. nucleatum, P. gingivalis*) and beneficial oral bacteria (*Weissella cibaria*) showed that *F. nucleatum* reduced the number of viable cells up to 1.5 10^8^ CFU/mL, and *P. gingivalis* reduced the number of viable cells to < 10^5^ CFU/mL, compared to controls [19]. In this experiment *L. gasseri* HHuMIN D inhibited *F. nucleatum* more effectively. Additionally, *L. gasseri* HHuMIN D inhibited *P. intermedia*, *P. catoniae* and seven facultative anaerobic bacteria quite effectively.

In another study, *Lactobacillus salivarius*, *Lactobacillus fermentum,* and the fermentation broths of these bacteria showed a definite inhibitory effect against harmful periodontal bacteria. *Lactobacillus* spp. showed greater direct antibacterial effects against harmful oral bacteria than their microbial supernatants. As the number of lactic acid bacteria and the concentration of the fermentation broth increased, the antibacterial effect also increased [48]. The current research results are similar to those of Chen et al. (2012): *L. gasseri* HHuMIN D's antibacterial effect was greater than *L. gasseri* HHuMIN D's supernatant antibacterial effect. This indicates the direct inhibition of harmful bacteria by *L. gasseri* HHuMIN D and indirect inhibition by the metabolites it produces [21, 43]. *L. gasseri* HHuMIN D strongly inhibited all harmful oral bacteria, but the supernatant of this bacteria inhibited only anaerobic oral bacteria and had different effects. The combination of these two actions efficiently kills pathogenic microorganisms in the oral environment [43]. *L. gasseri* HHuMIN D may, therefore, mitigate oral diseases caused by harmful oral bacteria.

### Accumulation of hydrogen peroxide by *L. gasseri* HHuMIN D

**Fig. 3:**
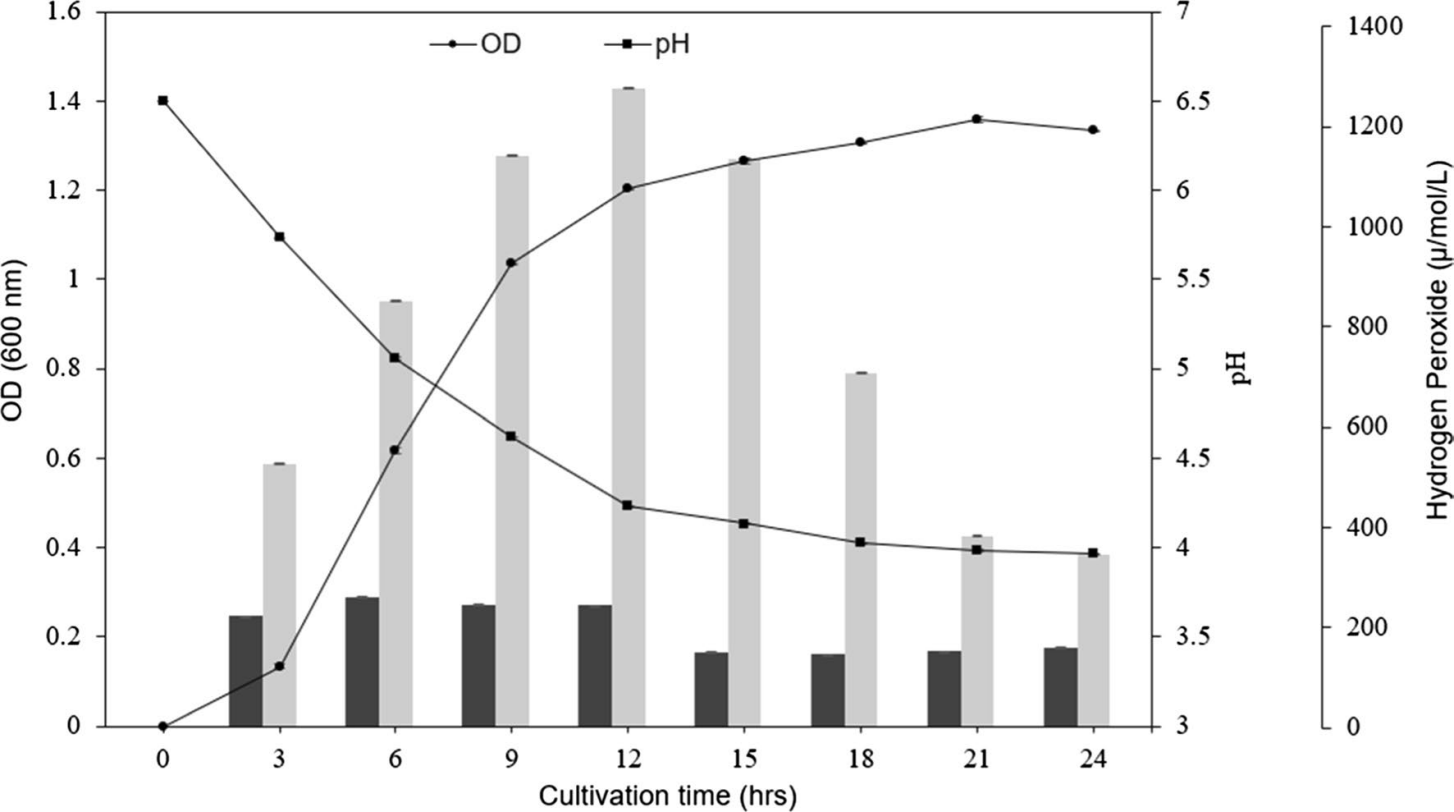
Optical density (●), pH (■) and accumulation of hydrogen peroxide of *L. gasseri* HHuMIN D (light gray) and *W. cibaria* (dark gray) cultivated in MRS medium (pH6.5) at 37℃ under anaerobic conditions (n = 3)

Oral *Lactobacillus* spp. produces bacteriocins, lactic acid and hydrogen peroxide, which act as a defense against pathogens [22, 49]. The generation of hydrogen peroxide is a typical function of *Lactobacillus* spp. (*L. bulgaricus, L. lactis*, and *L. plantarum*) isolated from the oral cavity. Several studies have shown that these beneficial bacteria inhibit the growth of pathogenic microorganisms such as *Staphylococcus aureus* [50]*, Pseudomonas* spp*.* [51], and psychrotrophic bacteria [52, 53]. The hydrogen peroxide produced by *L. gasseri* HHuMIN D increased continuously from 0 μmol/L to 802 μmol/L after 3 h (Fig. 3). After 12 h, the concentration of hydrogen peroxide decreased steadily to 24 h. *L. johnsonii* and *L. gasseri* have been shown to produce hydrogen peroxide at rates between 400 and 1400 μmol/L [54]. Other research found that *S. sanguis* released hydrogen peroxide up to 30 μmol/L, *S. oralis* released hydrogen peroxide up to 640 μmol/L and both inhibited plaque formation by inhibiting *S. mutans* proliferation. The application of lactoperoxidase and thiocyanate to oxygen-supplied cultures revealed that *S. mutans* was inhibited, but did not affect the growth of, *S. sanguis* and *S. oralis* [55]. Additional research contends that H_2_O_2_ produced by beneficial bacteria can decrease halitosis by inhibiting the growth of *F. nucleatum* and reducing the VSC produced by oral anaerobic bacteria [56].

*L. gasseri* HHuMIN D was shown to produce good amounts of hydrogen peroxide and is predicted to suppress harmful anaerobic bacteria that are vulnerable to hydrogen peroxide by continuous production. Further experimentation will be necessary to specifically investigate the effect of *L. gasseri* HHuMIN D's H_2_O_2_ on oral anaerobic bacteria.

### Susceptibility of the bacteriocin produced by *L. gasserie* HHuMIN D to hydrolytic enzymes

Antibacterial activity was completely lost by proteinase K, trypsin, and α-chymotrypsin treatment, indicating that the antibacterial substance produced by *L. gasseri* HHuMIN D is proteinaceous, and possibly bacteriocin (Table 3). The fact that the activity was not lost by pepsin treatment is possibly an enzyme concentration issue or indicative of the lack of a specific cleavage site for pepsin to react at. Since α-amylase did not affect the antibacterial activity, the carbohydrate moiety was either not present in the antibacterial molecule or not related to the antibacterial activity. Bacteriocins in which antibacterial activity is lost only with proteolytic enzyme treatment have previously been reported, as in the case of *Bacillus licheniformis* [27]. The greatest advantage of bacteriocin is that it is composed of proteins or peptides and it is decomposed by proteolytic enzymes in the digestive tract of the human body, so it is considered to be non-toxic and non-persistent [57–60].

**Table 3:** Susceptibility of *L. gasserie* HHuMIN D culture supernatant antimicrobial activity to various hydrolytic enzymes

Enzyme	Activity
Control (no-enzyme)	+++
Proteinase K	−
Trypsin	−
α-Chymotrypsin	−
Pepsin	+++
α-Amylase	+++

*Fusobacterium nucleatum, Porphyromonas gingivalis, Prevotella intermedia,* and *Porphyromonas catoniae* were used as indicators. Degree of growth inhibition by OD (600 nm): “+++”, value similar to control; “++”, value weaker than control; “−”, no growth. Culture supernatant was mixed with enzyme solutions at a final concentration of 1 mg/ml

### Coaggregation of *L. gasserie* HHuMIN D and oral microorganisms

*L. gasseri* HHuMIN D was shown to have the highest cohesion with *P. catoniae* (70%) and *P. intermedia* (28 ± 5%) and there was no cohesion with *P. gingivalis* or *F. nucleatum.* Amongst aerobic oral bacteria, *L. gasseri* HHuMIN D coaggregated with *S. sanguinis* best (74 ± 2%), followed by *S. gordonii* (62 ± 1%), *S. mutans* (49 ± 4%), *S. mitis* (46 ± 3%), *S. sobrinus* (37 ± 2%), *S. oralis* (30 ± 2%), and *S. parasanguinis* (6 ± 3%). However, the coaggregation of *W. cibaria* was relatively weaker than that of *L. gasseri* HHuMIN D (Table 4). *L. gasseri* HHuMIN D generally has higher coaggregation with oral facultative anaerobic bacteria than oral anaerobic bacteria with the exception of *P. catoniae*, which indicates that *L. gasseri* HHuMIN D possesses effective inhibitory capacity against oral facultative anaerobic bacteria. In another study evaluating the accumulation of 4 strains of human streptococci and 6 strains of lactobacillus used in consumer products, all probiotic strains showed the ability to aggregate with oral pathogens, but the degree of aggregation was different for each strain and dependent on time [61]. This indicates that only some *Lactobacillus* strains can aggregate with harmful bacteria.

**Table 4:** Coaggregation reactions between *W.* cibaria or L*. gasseri* HHuMIN D and various periodontal bacteria

Strains	Coaggregation (%)	
	*W. cibaria*	*L. gasseri* HHuMIN D
*Fusobacterium nucleatum* KCOM 1001	4 ± 2^a^	2 ± 2^a^
*Porphyromonas gingivalis* KCOM 2796	0 ± 1^a^	0^a^
*Prevotella intermedia* KCOM 2889	0 ± 2^a^	28 ± 5^b^
*Porphyromonas catoniae* KCOM3169	0 ± 1^a^	70 ± 0^c^
*Streptococcus sobrinus* KCOM1157	20 ± 1^b^	37 ± 2^b^
*Streptococcus mitis* KCOM 1356	2 ± 0^a^	46 ± 3^d^
*Streptococcus oralis* KCOM 1493	0 ± 1^a^	30 ± 2^b^
*Streptococcus gordonii* KCOM 1788	11 ± 6^b^	62 ± 1^c^
*Streptococcus sanguinis* KCOM 2167	50 ± 3^c^	74 ± 2^e^
*Streptococcus parasanguinis* KCOM 2522	0 ± 3^a^	6 ± 3^a^
*Streptococcus mutans* KCTC3065	29 ± 4^d^	49 ± 4^d^

Values are expressed as the mean ± standard deviation. Means not sharing a common letter are significantly different groups at *p* < 0.001 (n = 3)

Over 500 bacterial species have been identified in the oral cavity, of which 15–20 are known to produce toxic materials and are directly implicated in the evolution of various forms of periodontal disease [62]. Many of these harmful bacteria are also highly capable of coagulating with other microorganisms, live in the oral cavity and create toxic substances constantly, thus harming oral health [63]. Although the mechanism of aggregation has not been identified, the ability of probiotics to coaggregate with harmful bacteria is important as a secondary function to support the main function of probiotics. Oral probiotics must adhere well with harmful oral bacteria, inhibit their growth, and effectively diminish oral disease [64, 65]. Additionally, harmful bacteria are exposed directly to antimicrobials such as bacteriocins, lactic acid, and hydrogen peroxide through direct coaggregation with beneficial bacteria [66]. *L. gasseri* HHuMIN D's ability to coaggregate with harmful bacteria is therefore an important indicator of its potential value as an oral probiotic.

### Effect of *L. gasseri *HHuMIN D on the formation of artificial dental plaque by *S. mutans*

**Fig. 4:**
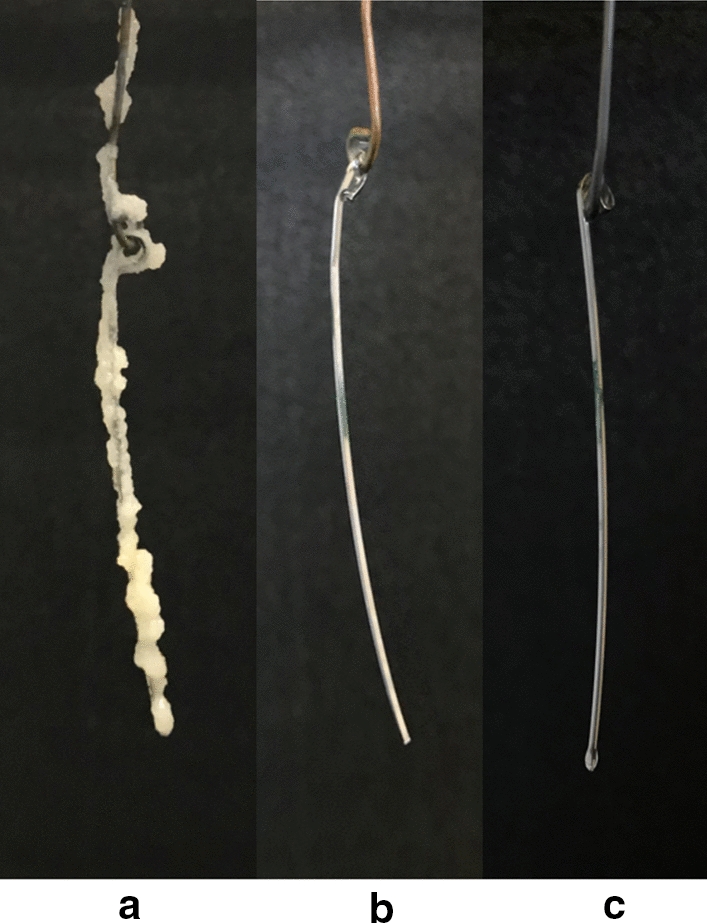
Effect of *L. gasseri* HHuMIN D on the formation of artificial plaque by *S. mutans* on wires using BHI media containing 5% sucrose. Artificial plaque formed on wires in *S. mutans* single culture (**a**), *L. gasseri* HHuMIN D single culture (**b**) and *L. gasseri* HHuMIN D / *S. mutans* co-culture culture (**c**) are shown

Artificial dental plaques formed on calibration wire suspended in *S. mutans* inoculated broths, but such plaques were not developed in *L. gasseri* HHuMIN D and *W. cibaria* inoculated broths. No artificial dental plaque was formed on calibration wire in broth co-cultured with *S. mutans* and *L. gasseri* HHuMIN D, indicating 100% inhibition (Fig. 4; Table 5).

**Table 5:** Effect of *L. gasseri* HHuMIN D on the formation of artificial plaque by* Streptococcus mutans* on wires using BHI media containing 5% sucrose

Tested bacterial strain	Plaque weight (mg)
*S. mutans*	105 ± 5^a^
*W. cibaria*	0 ± 0^b^
*L. gasseri* HHuMIN D	0 ± 0^b^
*W. cibaria* + *S. mutans*	53.4 ± 12^c^
*L. gasseri* HHuMIN D + *S. mutans*	0 ± 0^b^

The values are expressed as the mean ± standard deviation. Means not sharing a common letter are significantly different groups at *p* < 0.001 (n = 3)

In one study, *S. mutans* created artificial dental plaque was suppressed by 53% by *E. faecium* T7 co-culture [29]. In another experiment, *Lactobacillus* lactis 1370 suppressed plaque formation by 95% [67]. Compared to other organisms tested, *L. gasseri* HHuMIN D strongly inhibits dental plaque formation by *S. mutans*.

### Adhesion of *L. gasseri HHuMIN D*

Oral epithelial cell monolayer testing is a method used to identify beneficial bacteria and has also been used as a guide to test the attachment of beneficial bacteria to the oral epithelium. According to an earlier study, if the number of attached bacteria per KB cell is 1.5 or more, the attachment capacity is considered to be very strong and if the number of attached bacteria is 1.5 to 1, the adhesion capacity is considered to be strong. If the number of bacteria attached is 1 to 0.5, the ability to adhere is moderate and if the number of the bacteria attached is less than 0.5, the ability to adhere is considered weak [68]. The KB cell adhesion ability of *L. gasseri* HHuMIN D was determined to be 4.41 ± 1.4 cells per cell– very strong. *F. nucleatum* showed a very high adhesion capacity of 18.35 ± 4.0, and *S. mutans* showed a strong adhesion capacity of 2.57 ± 0.1 (Table 6). Strains with strong adhesion are likely to adhere to oral epithelial cells, form colonies, and inhibit the attachment of pathogenic bacteria to epithelial cells. In a previous analysis, the adhesion abilities of harmful bacteria were measured for *P. intermedia* from 4.110^4^ ± 2.710^4^ to 152 ± 57 per 10^5^ KB cells, for *P. gingivalis* at 9.610^5^ ± 1.010^5^, and for *E. coli* at 278 ± 133 [68]. In another study, *F. nucleatum’*s KB cell adhesion at the initial dose was from 19.2 ± 0.3% to 1.5 ± 0.4, suggesting that *F. nucleatum* has very strong adhesion capabilities [69].

**Table 6:** Adhesion ability to KB cells of tested strains

Strains	No. of CFU recovered
*L. gasseri* HHuMIN D	4.41 ± 1.4^a^
*F. nucleatum* KCOM1001	18.35 ± 4.0^b^
*S. mutans* KCTC3065	2.57 ± 0.1^a^

The values are expressed as the mean ± standard deviation for triplicate samples of lysates from the infection of 10^5^ KB cells by 10^8^ bacteria. Means not sharing a common letter are significantly different groups at p < 0.01 (n = 3)

The ability of *L. gasseri* HHuMIN D to inhibit cell adhesion by harmful bacteria was assessed by conducting protection and displacement assays. In the protection assays, *L. gasseri* HHuMIN D was allowed to bind to KB cells before introducing harmful oral bacteria. *L. gasseri* HHuMIN D reduced the adhesion of *F. nucleatum* and *S. mutans* to cells by 100% and 90%, respectively. Unfortunately, *L. gasseri* HHuMIN D’s cell adhesion was reduced by 63% and 71%, respectively, by competition with *F. nucleatum* and *S. mutans*. *L. gasseri* HHuMIN D strongly inhibited *F. nucleatum* adhesion and it appears that the cell adhesion of *L. gasseri* HHuMIN D might also have been reduced by competition with harmful oral bacteria (Table 7).

**Table 7:** The number of experimental bacteria and pathogens bound to KB cells in adhesion ability, protection assays and displacement assays

	Strains	No. of CFU recovered ^a^ (× 10^5^)	Adhesion inhibitory rate (%)
Positive Control	*L. gasseri* HHuMIN D	4.41 ± 1.4	
	*F. nucleatum* KCOM1001	18.35 ± 4.0	
	*S. mutans* KCTC3065	2.57 ± 0.1	
Protection assays^b^	(*L. gasseri* HHuMIN D → *F. nucleatum* KCOM1001) *L. gasseri* HHuMIN D	1.64 ± 0.2**	63
	(*L. gasseri* HHuMIN D → *F. nucleatum* KCOM1001) *F. nucleatum* KCOM1001	0.05 ± 0.0***	100
	(*L. gasseri* HHuMIN D → *S. mutans* KCTC3065) *L. gasseri* HHuMIN D	1.27 ± 0.6**	71
	(*L. gasseri* HHuMIN D → *S. mutans* KCTC3065) *S. mutans* KCTC3065	0.25 ± 0.2****	90
Displacement assays^c^	(*F. nucleatum* KCOM1001 → *L. gasseri* HHuMIN D) *L. gasseri* HHuMIN D	1.32 ± 0.4**	70
	(*F. nucleatum* KCOM1001 → *L. gasseri* HHuMIN D) *F. nucleatum* KCOM1001	2.10 ± 0.8***	89
	(*S. mutans* KCTC3065 → *L. gasseri* HHuMIN D) *L. gasseri* HHuMIN D	1.18 ± 0.3**	73
	(*S. mutans* KCTC3065 → *L. gasseri* HHuMIN D) *S. mutans* KCTC3065	0.27 ± 0.1****	90

^a^Values represent the means ± the standard deviations for triplicate samples of lysates from the infection of 10^5^ KB cells by 10^8^ bacteria. Compared with the positive control group: *p < 0.05, **p < 0.01, ***p < 0.001, ****p < 0.0001 (n = 3)

^b^Protection assays are a method of investigating how harmful bacteria are inhibited from attaching by selected bacteria which are already attached

^c^Displacement assays are a method of investigating the degree of attachment by selected bacteria after harmful bacteria are already attached

The attachment of *L. gasseri* HHuMIN D after the attachment of harmful oral bacteria was measured in displacement assays. With *F. nucleatum* and *S. mutans*, the cell adhesion of *L. gasseri* HHuMIN D decreased to 70 and 73%, respectively. However, the attachment of harmful oral bacteria decreased by 89 and 90%, respectively. Taken together, these two results indicate a stronger ability to inhibit the binding of harmful bacteria when first bound to *L. gasseri* HHuMIN D. This suggests that *L. gasseri* HHuMIN D is more effective in preventing than inhibiting the attachment of harmful oral bacteria. Consequently, continuous intake is likely necessary to enable adhesion of *L. gasseri* HHuMIN D to highly concentrated oral cavity cells. Other researchers have theorized there is competition for common adhesion receptors between harmful bacteria and beneficial oral bacteria [70] and that antibacterial or antiadhesive factors produced by beneficial bacteria inhibit harmful oral bacteria from adhering after beneficial bacteria aggregate [71].

### Safety evaluations of *L. gasseri* HHuMIN D

Microorganisms may create various poisonous substances by nitrogen derivatives through protein, peptide and amino acid decomposition in saliva or food [73]. When a microorganism enters the large intestine, it is able to generate poisonous substances such as phenol, ammonia and indole by decomposition of proteins [72]. Ammonia formed by microorganisms is known to migrate to the liver and cause cell damage cofactors and chronic hepatic damage. The production of ammonia from microorganisms is closely related to human health and must be assessed to demonstrate the safety of commercial probiotics. According to Vince and Burridge [73], *Clostridia, Enterobacter, Bacillus* spp., and Gram-negative anaerobes create large amounts of ammonia. Furthermore, certain *Streptococci, Micrococci*, and Gram-positive non-spore forming anaerobes release small quantities of ammonia, and Gram-positive aerobic rods generate trace amounts of ammonia. Certain strains of *Lactobacillus* can produce small amounts of ammonia during growth. The evaluation of ammonia production confirmed the safety of *L. gasseri* HHuMIN D; it produced no ammonia. In contrast, *Enterococcus faecium* ATCC19433, a positive control, produced 109 ± 7 μg/mL of ammonia. *L. gasseri* HHuMIN D's ammonia production is below the level of concern in South Korea's Ministry of Food and Drug Safety's milk product quality [23].

Biogenic amines (BAs) derived from amino acids are common anti-nutritional compounds in animals and humans. Ingestion of massive amounts of BAs may cause symptoms similar to significant allergic reactions. BAs have been identified as causative agents in many cases of food poisoning and are critical from a hygienic point of view because they can induce a variety of pharmacological reactions [74]. BAs are involved in numerous mammalian metabolic and intracellular processes, such as synaptic transmission, modulation of blood pressure, allergic reactions, and management of cellular growth. Probiotic bacteria, commonly used in the food industry, produce BAs through microbial metabolic activities such as decarboxylation and protein molecule transamination [75]. *L. gasseri* HHuMIN D did not produce cadaverine, histamine, putrescine, or tyramine. Since ammonia and/or BAs are used as a quality indicator for fermented foods, *L. gasseri* HHuMIN D's absence of ammonia and BA activity suggests that *L. gasseri* HHuMIN D is suitable for use in the manufacture of fermented and non-fermented foods.

**Fig. 5:**
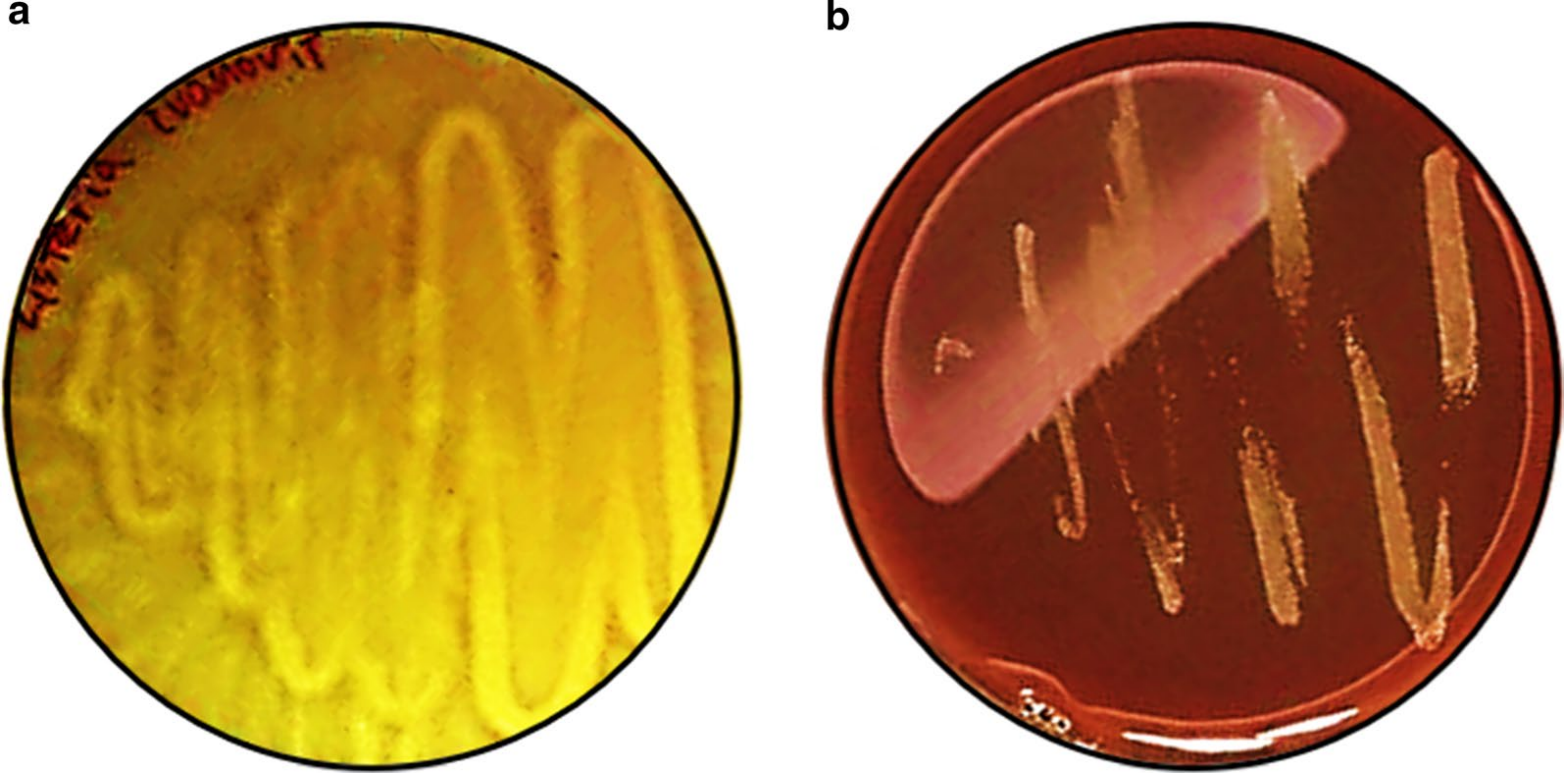
Hemolysis activity of *L. ivanovii* subsp. *ivanovii* ATCC 19,119 (**a**); positive control, back light) (beta hemolytic) and *L. gasseri* HHuMIN D (**b**); back light). Purified culture strains were streaked on 5% blood agar plate. After 24 h, the changes of plate color were observed. *L. gasseri* HHuMIN D growth indicated no blood cell lysis

The *Guidelines for the Evaluation of Probiotics in Food*, produced by FAO and WHO joint research, states, “If the strain under evaluation belongs to a species with known hemolytic potential, determination of hemolytic activity is required” [24, 76]. The hemolytic characteristics of microorganisms are an important measurement criterion for the safety of bacteria since they may liquefy/degrade red blood cells and ultimately cause anemia and edema. Among probiotics, *Lactobacillus* spp. are graded as α-hemolytic microorganisms [77]. However, several *Lactobacillus* spp. (*L. sakei* MBSa1 bac + , *L. curvatus* MBSa3 bac + and *L. lactis* 368 bac–) demonstrate strong β-hemolysis [78]. *L. ivanovii* developed β-hemolysis colorless zones around colonies in BL agar added 5% sheep blood but *L. gasseri* HHuMIN D cultivated in the same medium did not reveal colorless zones around the colonies (Fig. 5). Therefore, *L. gasseri* HHuMIN D does not cause hemolysis.

While there is no need for a safety assessment for mucin deterioration in the oral cavity, this study was performed to take into consideration the potential for ingestion and entry to the intestine by *L. gasseri* HHuMIN D. *L. gasseri* HHuMIN D was also assessed for the potential of translocation by in vitro mucolytic assays. Several research teams have studied the mucous dissolving capacities of human pathogenic bacteria since 1980, and that is now considered a measure of microbial virulence and microbial toxicity [79–82]. The intestinal mucus gel coating is a membrane made of glycoproteins and is an essential part of the intestine. Bacterial translocation is considered one of the most critical probiotic safety tests due to the risk for septicemia and bacteremia endocarditis [83]. Although most microorganisms do not exhibit mucolytic activity, several studies have reported that certain microorganisms do and the genes that induce mucin degradation enzymes have been identified [84]. Various intestinal pathogens are known to hydrolyze glycoprotein-based mucus gel layers and possess the ability to metabolize mucus-derived monosaccharides [85].

**Fig. 6:**
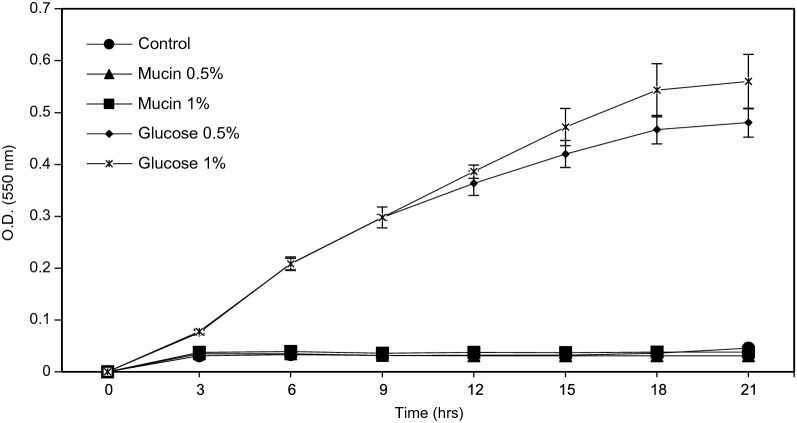
The effect of different concentrations (0, 0.5 and 1% [w/v]) of mucin and dextrose on *L. gasseri* HHuMIN D in modified MRS, evaluated by optical density (550 nm) and recorded after 3 to 21 h (n = 3)

If *L. gasseri* HHuMIN D had the ability to produce mucinases, through mucin degradation it would be able to survive and grow aggressively in the presence of mucin and no other carbohydrate sources. As illustrated in Fig. 6, *L. gasseri* HHuMIN D growth was actively induced by the addition of glucose as the carbon source. But growth was not observed when glucose was replace by mucin. These results show that *L. gasseri* HHuMIN D does not use mucin as a carbon source for growth.

To distinguish antibiotic tolerance from antibiotic-sensitivity, microbiological cut-off values for the antibiotic tolerance of microorganisms used as food were defined by the European Food Safety Authority [86]. In a study of *Lactobacillus* spp.'s susceptibility to 23 antibiotics, some lactobacilli showed resistance to kanamycin, vancomycin, and chloramphenicol. The authors hypothesized that strains with these genes did not necessarily indicate cause for concern about the transition of antibiotic resistance and could be used in food and medicinal formulations as natural biopreservatives [87]. Ampicillin, erythromycin, vancomycin, chloramphenicol, and clindamycin were low compared to the cut-off values proposed by EFSA. Antibiotic resistance can be transmitted via plasmids, the assessment of antibiotic resistance is a significant criterion for assessing *Lactobacillus* sp. safety [88]. Therefore, we used WGS to genetically identify the antibiotic resistance gene of *L. gasseri* HHuMIN D and ascertain the possibility of transmission to other bacteria through the presence or absence of a plasmid. Except for gentamicin, streptomycin, and kanamycin, the MIC values of *L. gasseri* HHuMIN D were less than or equal to the cut-off values proposed by the EFSA. As shown in Table 8, *L. gasseri* HHuMIN D was sensitive to ampicillin, carbenicllin, cephalothin, chloramphenicol, clindamycin, dicloxacillin sodium salt hydrate, erythromycin, lincomycin, methicillin, penicillin G, tetracycline, and vancomycin (MICs ranged from 0.01 to 4 µg/mL). There was general resistance to bacitracin, gentamicin, katamycin, metronidazole, neomycin, polymyxin B, phosphomycin, streptomycin, and trimethoprim-Sulfamethoxazole (all MICs were greater than 32 µg/mL).

**Table 8:** Antimicrobial susceptibility (MIC values) of *L. gasseri* HHuMIN D

Classification	Antimicrobial agent	(MIC values, µg/mL)			
		Cut-off of *Lactobacillus* (EFSA^a^)		Strain	
		*Lactobacillus* obligate homofermenta tive^b^	*Lactobacillus* obligate heterofermentative^c^	*L. gasseri* HHuMIN D	*E. faecium* ATCC 29,212
β-Lactam group	Penicillin G			0.063	0.5
	Carbenicllin (disodium salt)			0.5	8
	Methicillin			2	16
	Ampicillin (sodium salt)	1	2	0.25	0.25
	Dicloxacillin sodium salt hydrate			0.5	4
Aminoglycoside group	Gentamicin (sulfate)	16	16	32	256
	Streptomycin (sulfate salt)	16	64	64	> 256
	Kanamycin (sulfate)	16	32	512	256
	Neomycin (sulfate)			256	1024
Cephem group	Cephalothin (sodium salt)			1	16
Tetracycline group	Tetracycline	4	4	4	32
Peptide group	Polymyxin B (sulfate salt)			> 1024	> 1024
	Bacitracin			64	
Macrolide group	Erythromycin	1	1	0.5	8
Synthetic antimicrobial group	Metronidazole			> 256	> 256
The other group	Vancomycin (HCl)	2^d^	n.r	0.5	2
	Chloramphenicol	4	4	2	8
	Lincomycin (hydrochloride)			4	
	Rifampicin			< 0.125	0.5
	Clindamycin (hydrochloride)	1	1	0.5	> 16
	Phosphomycin (disodium salt)			> 1024	32
	Mupirocin			16	64
	Trimethoprim-Sulfamethoxazole (Co-trimoxazole)			> 256	32

*n.r.* not required.

^a^Data from EFSA (2012)

^b^including *L. delbrueckii*, *L. helveticus*, *L. gasseri*

^c^including *L. fermentum*

^d^not required for *L. salivarius*

**Fig. 7:**
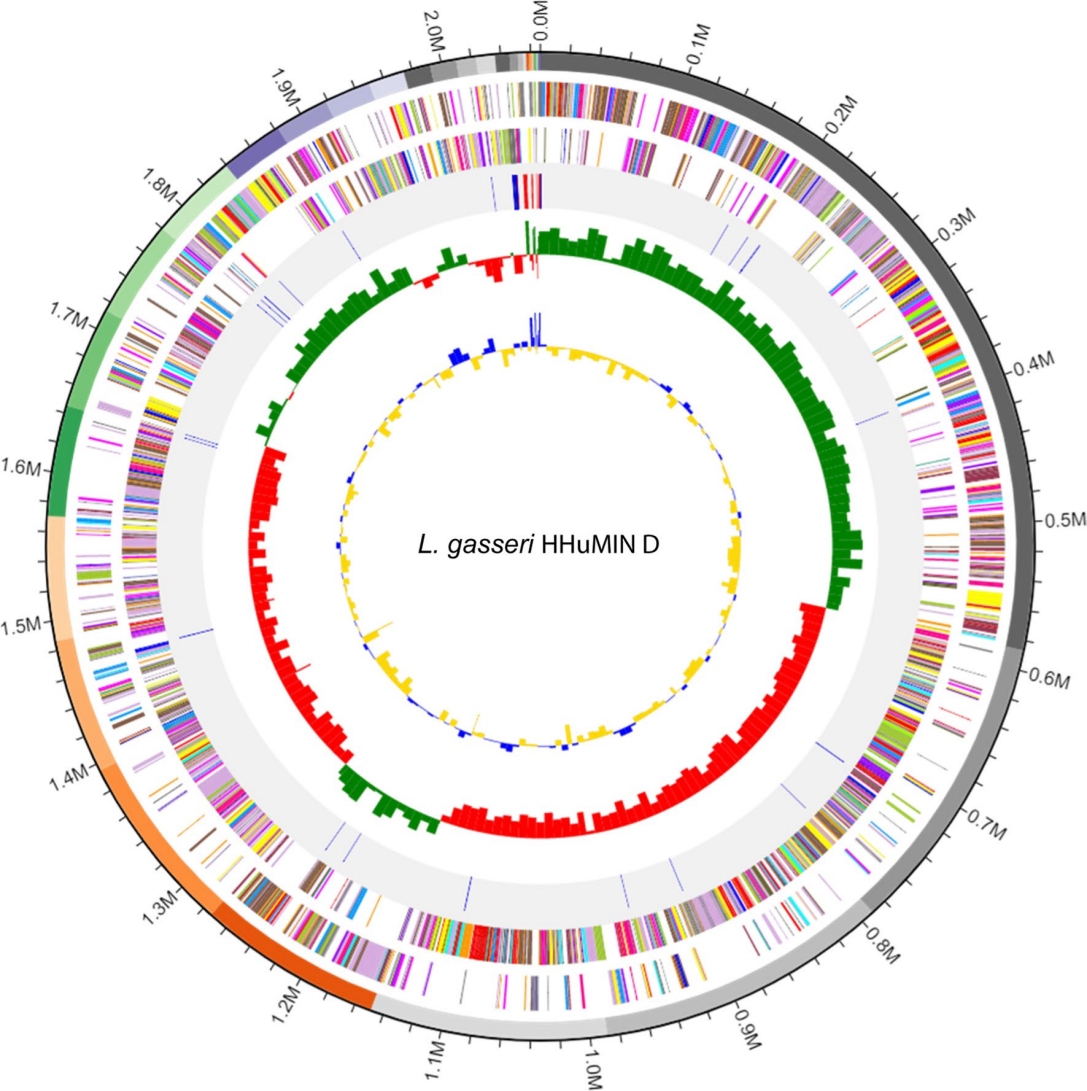
Genome map of *L. gasseri* HHuMIN D. Whole genome sequencing of *L. gasseri* HHuMIN D was performed by Chunlab, Inc. (Seoul, Korea), which used PacBio Sequel Systems (Pacific Biosciences, Menlo Park, CA, USA) and analyzed using CLgenomics™ and EZBioCloud Apps programs (Chunlab, Seoul, Korea). The gene content circular image consists of five circles, and each circle shows the information of rRNA/tRNA, Reverse CDS, Forward CDS, GC Ratio and GC skew from the outside to the inside

**Fig. 8:**
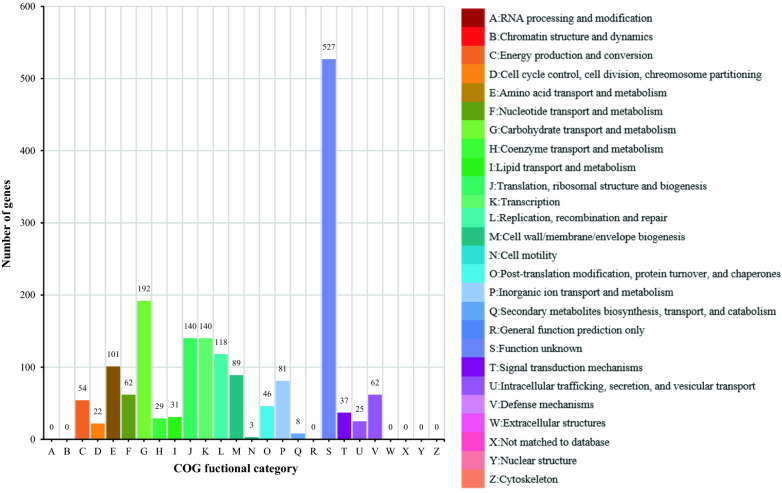
Functional categories based on EggNog/COG of genome of *L. gasseri* HHuMIN D. Whole genome sequencing of *L. gasseri* HHuMIN D was performed by Chunlab, Inc. (Seoul, Korea), which used PacBio Sequel Systems (Pacific Biosciences, Menlo Park, CA, USA) and analyzed using CLgenomics™ and EZBioCloud Apps programs (Chunlab, Seoul, Korea). The function of each protein was found through homology comparison with NCBI's RefSeq Database, and functional categories were completed by synthesizing information such as COG classification by NCBI COG, SEED classification by FIGfam, and EC classification etc. The figure is a combination of the assignments of each COG and the functional category (25 categories, which are often designated from A to Z) using EggNog

Whole genome sequencing (WGS) is a technique which studies the functional aspects of a microorganism by sequencing a microorganism's entire genome and comparing it to a gene previously identified [89]. The size of the entire gene sequence of *L. gasseri* HHuMIN D was 2,066,663 bp and the GC composition ratio was 34.9%. The average GC content of *Lactobacillus* spp. is 46.61%, *L. gasseri* HHuMIN D is lower than the average GC content of *Lactobacillus* spp. The number of rRNA genes and tRNA genes were 7 and 63, respectively. The number of coding sequences (CDSs) was 2,015, and the average of the coding sequence length was 923.9 bp. Figures 7 and 8 show a genetic map of *L. gasseri* HHuMIN D and a functional classification based on COG.

Bacteriocin is a proteinaceous bacterial product with bactericidal activity [90]. Any Gram-positive bacteria that produce bacteriocin are considered to be active against Gram-negative bacteria [91]. This broad spectrum of compounds is important in preventing the growth of harmful bacteria and alleviating disease. They are produced by specific lactic acid bacteria, including lactococci, lactobacilli, and pediococci [92].

Since 1980, the *L. acidophilus* group has been classified by DNA-DNA homology into six homologous species: *L. acidophilus*, *L. amylovorus*, *L. crispatus*, *L. gallinarum*, *L. gasseri* and *L. johnsonii* [93, 94]. Out of these six species *L. gasseri* is thought to be the primary member inhabiting the human intestine, and studies on the bacteriocins produced by this bacterium have been conducted [94–96]. Lactacin F is the best-studied bacterocin in the LAB class IIb. Gassericin T is a 2-component bacteriocin consisting of GatA and GatX and of the lacticin-F family; it is known as the primary bacteriocin developed by *L. gasseri* strains [97]. Gassericin T is heat-stable (121 °C, 10 min), pH-tolerant (pH 2–11) and bactericidal against several food poisoning gram-positive bacteria such as *Bacillus cereus, Listeria monocytogenes, and Staphylococcus aureus* [94]. Helveticin J was first studied in *L. helveticus*. Helveticin J can be used as an indicator of closely related species, is active at neutral pH under aerobic or anaerobic conditions and is sensitive to proteases and heat (30 min at 100 °C) [98]. These 2 bacteroicins are good candidate biopreservatives.

The whole genome sequence of *L. gasseri* HHuMIN D was verified through CLgenomicsTM and EZBioCloud applications to see if this strain qualifies for GRAS status. All results are summarized in Table 9. We found that the antimicrobial activity against oral harmful bacteria may be the result of one or more of three bacteriocin genes in which the antimicrobial activity of *L. gasseri* HHuMIN D was found: hlv, lafA, lafX. The helveticin J from *L. gasseri* HHuMIN D showed 98.09% homology with helveticin from *L. paragasseri* strain NCTC13720, and 97.89% homology with helveticin from *L. gasseri* strain EJL. The lactacin-F (subunit LafA and subunit LafX) from *L. gasseri* HHuMIN D showed 100% homology with lactacin-F from *L. gasseri* strain EJL and *L. gasseri* strain 151–2. The WGS cannot determine which of these genes are responsible for inhibitory activity against the pathogens tested and there is no data for reference because there are insufficient screening studies on bacteriocins produced by *L. gasseri* HHuMIN D against harmful oral bacteria. Further studies on bacteriocins produced by *L. gasseri* HHuMIN D and their effects on harmful oral bacteria are needed.

**Table 9:** Genes and genetic characteristics of *L. gasseri* HHuMIN D bacteriocin on the basis of whole genome sequencing results

Gene	Length	Product	Database hit	Homology
hlv	996	Bacteriocin helveticin-J	UniProtKB: P22294	*L. paragasseri* strain NCTC 13,720 (98.09%), *L. gasseri* strain EJL (97.89%)
lafA	228	Bacteriocin lactacin-F subunit LafA	UniProtKB: P24022 NCBI: LJ_RS03150	*L. gasseri* strain EJL (100%), *L. gasseri* strain 151–2 (100%)
lafX	198	Bacteriocin lactacin-F subunit LafX	UniProtKB: Q48509	*L. gasseri* strain EJL (100%), *L. gasseri* strain 151–2 (100%)

**Fig. 9:**
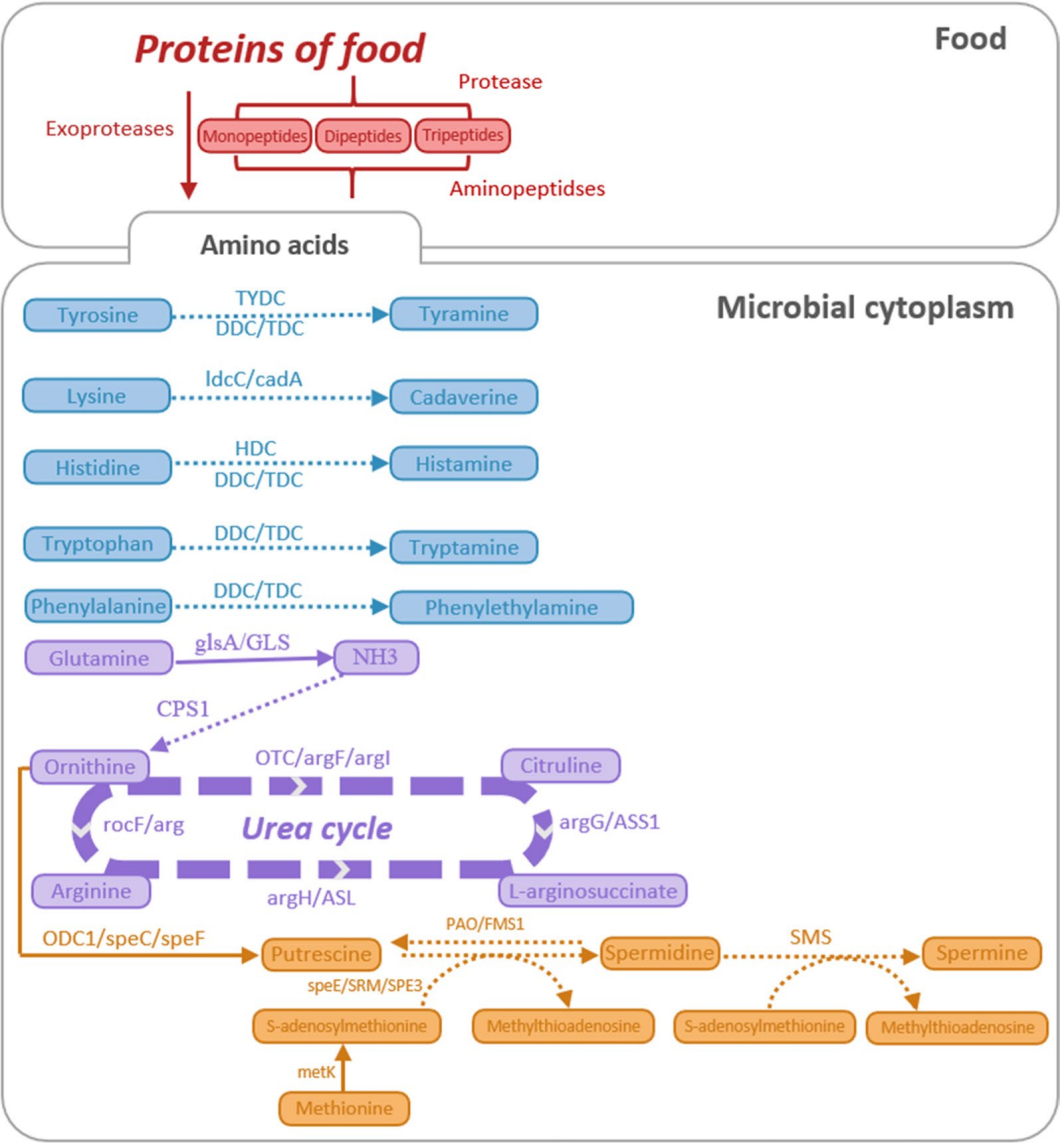
Biosynthetic pathways of different biogenic amines by *L. gasseri* HHuMIN D. Solid arrows indicate that the biosynthesis pathways of biogenic amine is effective and dashed arrows indicate invalid: *TYDC* tyrosine decarboxylase, DDC/TDC L-tryptophan decarboxylase, *ldcC/cadA* lysine decarboxylase, *hdc* HDC, histidine decarboxylase, *glsA/GLS* glutaminase, *CPS1* carbamoyl-phosphate synthase, *OTC/argF/argI* ornithine carbamoyl transferase, *argG/ASS1* argininosuccinate synthase, *argH/ASL* argininosuccinate lyase, *rocF/arg* arginase, *ODC1/speC/speF* ornithine decarboxylase, *PAO/FMS1* polyamine oxidase, *SMS* spermine synthase, *speE/SRM/SPE3* spermidine synthase, and *metK* S-adenosylmethionine synthetase. Whole genome sequencing of *L. gasseri* HHuMIN D was performed by Chunlab, Inc. (Seoul, Korea), which used PacBio Sequel Systems (Pacific Biosciences, Menlo Park, CA, USA) and analyzed using CLgenomics™ and EZBioCloud Apps programs (Chunlab, Seoul, Korea). Biosynthetic pathways of different biogenic amines were analyzed by matching with the KEGG pathway using complete genome sequencing of *L. gasseri* HHuMIN D via CL Genomics™ (ChunLab, Seoul, South Korea, 2004). The activities of related genes and substances produced can be inferred through the biosynthetic pathways provided by KEGG

The whole genome sequence of *L. gasseri* HHuMIN D was scanned via the CLgenomicsTM and EZBioCloud applications for genes that generate enzymes engaged in the metabolic pathways that create biogenic amines. Figure 9 demonstrates the biosynthetic mechanism of different biogenic amines.

No genes were identified that produce tyrosine decarboxylase (which generates tyramine from tyrosine), L-tryptophan decarboxylase (which generates tyramine from tyrosine, histamine from histidine, tryptamine from tryptophan, and phenylethylamine from phenylalanine), lysine decarboxylase (which generates cadaverine from lysine), histidine decarboxylase (which generates histamine from histidine), carbamoyl-phosphate synthase (which generates ornithine from NH3), ornithine carbamoyl transferase (which generates citruline from ornithine), argininosuccinate synthase (which generates L-arginosuccinate from citruline), argininosuccinate lyase (which generates arginine from L-arginosuccinate), arginase (which generates ornithine from arginine), polyamine oxidase (which generates putrescine from spermidine), spermine synthase (which generates spermine from spermidine), spermidine synthase (which generates methylthioadenosine from S-adenosylmethionine) (Fig. 9), and genes were identified that produce glutaminase (which generates NH3 from glutamine), ornithine decarboxylase (which generates putrescine from ornithine), S-adenosylmethionine synthetase (which generates S-adenosylmethionine from methionine) (Fig. 9).

Glutamine can be converted to NH3 through glsA or GLS and converted NH3 can be converted to ornithine through CPS1. As a result of confirming the mechanism, *L. gasseri* HHuMIN D can be converted from glutamine to NH3 via glsA or GLS but cannot be converted to ornithine since there is no CPS1 thereafter. If ornithine exists, it can be converted via the urea cycle into citriuline, L-arginosuccinate, and arginine; the associated genes have not been found in the *L. gasseri* HHuMIN D genome. Polyamines such as putrescine, spermidine and spermine are produced using ornithine as the starting substrates through different pathways (Fig. 9). Except for methionine adenosyltransferase (MAT) and putrescine biosynthetic genes (ODC1, speC, speF), no genes encoding polyamine biosynthetic enzymes were found in the *L. gasseri* HHuMIN D genome. Contrary to the genetic results, the production amount seems to be very small, as bioamines are not measured in BA production capacity evaluation.

**Fig. 10:**
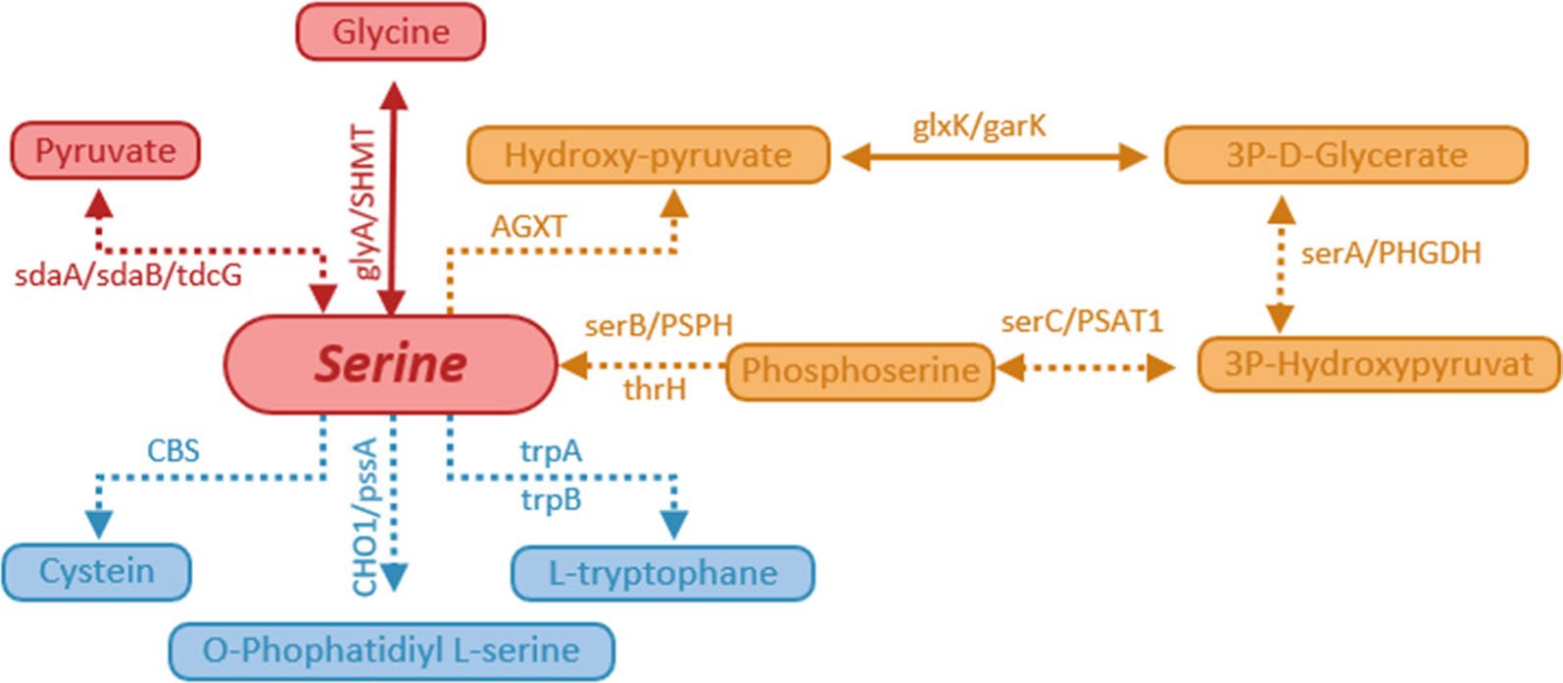
Glycine, serine, and threonine metabolism pathways of *L. gasseri* HHuMIN D. Solid arrows indicate that the metabolism pathways of glycine, serine, and threonine are effective and dashed arrows indicate invalid: *sdaA/sdaB/tdcG* L-serine dehydratase, *glyA/SHMT* glycine hydroxymethyltransferase, *AGXT* alanine-glyoxylate transaminase, *glxK/garK* glycerate 2-kinase, *serA/PHGDH* D-3-phosphoglycerate dehydrogenase, *serC/PSAT1* phosphoserine aminotransferase, *serB/PSPH* phosphoserine phosphatase, *thrH/phosphoserine* homoserine phosphotransferase, *CBS* cystathionine beta-synthase, *CHO1/pssA* CDP-diacylglycerol–-serine O-phosphatidyltransferase, *trpA* tryptophan synthase alpha chain; and trpB, tryptophan synthase beta chain. Whole genome sequencing of *L. gasseri* HHuMIN D was performed by Chunlab, Inc. (Seoul, Korea), which used PacBio Sequel Systems (Pacific Biosciences, Menlo Park, CA, USA) and analyzed using CLgenomics™ and EZBioCloud Apps programs (Chunlab, Seoul, Korea). Biosynthetic pathways of glycine, serine, and threonine were analyzed by matching with the KEGG pathway using complete genome sequencing of *L. gasseri* HHuMIN D via CL Genomics™ (ChunLab, Seoul, South Korea, 2004). The activities of related genes and substances produced can be inferred through the biosynthetic pathways provided by KEGG

Phosphatidylserine plays a crucial role in the development of blood clotting and thrombus, so the genes involved in the metabolic pathways that create this compound were evaluated. *L. gasseri* HHuMIN D does not have any genes that produce phosphatidylserine in the metabolic pathway (Fig. 10).

Virulence genes lend pathogenicity to microorganisms. *L. gasseri* HHuMIN D was assumed to be non-pathogenic using VirulenceFinder 2.0, a program that enables pathogenic and non-pathogenic bacteria to be differentiated using data from the whole genome sequence in order to classify potential virulence genes within the genome. The *L. gasseri* HHuMIN D genome sequence was compared with the genomic sequences of four noted pathogens (*Escherichia coli, Enterococcus, Listeria*, and *Staphylococcus aureus*). Virulence factors evaluated included *Escherichia coli* shiga toxin gene and *Staphylococcus aureus* exoenzyme genes, host immune alteration or evasion genes and toxin genes. The *L. gasseri* HHuMIN D genomic sequencing revealed no virulence factors or toxic or pathogenic genes.

*L. gasseri* HHuMIN D genes related to antibiotic resistance were discovered using the ideal and strict algorithms of CARD (Table 10). The genome of *L. gasseri* HHuMIN D was found to contain 16 putative genes associated with resistance to beta-lactams (7), bacitracin (1), aminoglycoside (1), aminocoumarin (1), lincomycin (1), polymyxin (1), macrolide (2) and multi antibiotic (2). Antibiotic resistance assays showed that *L. gasseri* HHuMIN D was resistant to gentamicin, streptomycin, and kanamycin, but genes associated with antibiotic resistance were not detected. Although penicillin, bacitracin, and lincomycin genes linked to resistance have been identified, the organism itself has demonstrated low resistance in antibiotic resistance assays. It must be noted that the *L. gasseri* HHuMIN D genome and the phenotype for antibiotic resistance do not match completely. It should be considered that the *L. fermentum* OK genome and the phenotypes of antibiotic resistance may not exactly fit. For DNA replication, GyrA is necessary. Multidrug efflux transporters are involved in several detoxifying activities in cells and are widely distributed across many forms of *Lactobacillus* spp. [89]. There are also safety factors regarding the use of antibiotic-resistant strains, due to the possibility of transferring antibiotic-resistance genes to intestinal pathogens [99]. Consequently, whole genome sequencing was used to determine whether the antibiotic-resistant genes of *L. gasseri* HHuMIN D could be transmitted through plasmids to other bacteria. No gene capable of delivering antibiotic resistance in the whole genome of *L. gasseri* HHuMIN D was found (data not shown). Since genes cannot be spread, antibiotic resistance to *L. gasseri* HHuMIN D is known to be inherent or normal. Several studies have documented that tolerance to aminoglycoside groups such as gentamicin, streptomycin, kanamycin and neomycin is suspected to be intrinsic to *Lactobacillus* spp. and is due to the absence of cytochrome-mediated electron transport that mediates drug absorption [100, 101].

**Table 10:** Putative antibiotic resistance genes in the *L. gasseri* HHuMIN D genome

Gene ID	Name	Length	COG_name	KEGG_name	Product	Category
HHuMIN D_00032	GLIA	1452	2814	22,134	multidrug resistance protein	multi antibiotic resistance gene
HHuMIN D_00185	cusR, copR, silR	666	0745	07,665	transcriptional regulatory protein	beta-lactam antibiotic resistance gene
HHuMIN D_00391	mdlB, smdB	1794	1132	18,890	probable multidrug resistance ABC transporter ATP-binding/permease protein YheH	multi antibiotic resistance gene
HHuMIN D_00573	aacC	810	2746	00,662	aminoglycoside N(3)-acetyltransferase	aminoglycoside antibiotic resistance gene
HHuMIN D_00588	mrcA	2379	0744	05,366	peptidoglycan glycosyltransferase	beta-lactam antibiotic resistance gene
HHuMIN D_00697	pbpB	2151	0768	08,724	penicillin-binding protein 2B	beta-lactam antibiotic resistance gene
HHuMIN D_00746	sotB	1383	0477	08,159	lincomycin resistance protein LmrB	lincomycin antibiotic resistance gene
HHuMIN D_00878	mrdA	2100	0768	05,515	serine-type D-Ala-D-Ala carboxypeptidase	beta-lactam antibiotic resistance gene
HHuMIN D_01245	gtrB, csbB	933	0463	20,534	undecaprenyl-phosphate 4-deoxy-4-formamido-L-arabinose transferase	polymyxin antibiotic resistance gene
HHuMIN D_01314	parE	1959	0187	02,622	DNA gyrase subunit	aminocoumarin antibiotic resistance gene
HHuMIN D_01342	abcA, bmrA	996	1132	18,104	xenobiotic-transporting ATPase	beta-lactam antibiotic resistance gene
HHuMIN D_01640	ddpF	678	1136	02,032	macrolide export ATP-binding/permease protein MacB	macrolide antibiotic resistance gene
HHuMIN D_01864	pbp1b	2829	0744	03,693	penicillin-binding protein 1A	beta-lactam antibiotic resistance gene
HHuMIN D_01995	ABC.CD.P	1869	0577	02,004	bacitracin export permease protein BceB	bacitracin antibiotic resistance gene
HHuMIN D_02034	cpoA	1044	0438	13,678	alpha-galactosylglucosyldiacylglycerol synthase	beta-lactam antibiotic resistance gene
HHuMIN D_02075	metN	690	1135	02,071	macrolide export ATP-binding/permease protein MacB	macrolide antibiotic resistance gene

## Conclusion

Oral microorganisms can be classified into anaerobic microorganisms and aerobic microorganisms (including facultative anaerobes), based on their sensitivity to oxygen. Anaerobic microorganisms generally live in environments where oxygen penetration is minimal or nonexistent, such as gingival crevices or periodontal pockets, and induce diseases such as halitosis and periodontitis. Aerobic microorganisms mainly inhabit the buccal and palatal mucosae, especially the tongue, and some species form biofilms to settle oral microorganisms and provide an environment in which diseases such as tooth decay can be induced. Most *Streptococcus* spp. do not induce disease and are commonly found in the oral cavity. We sought to confirm the effects of the probiotics isolated in this study using 11 types of oral microorganisms that included general aerobic microorganisms, disease-inducing aerobic microorganisms, and disease-inducing anaerobic microorganisms. *L. gasseri* HHuMIN D culture supernatant inhibited halitosis producing anaerobic microorganisms by 88.8% and antimicrobial activity against deleterious oral bacteria was strong. Multiplication of *L. gasseri* HHuMIN D was impaired when co-cultured with other oral bacteria. *L. gasseri* HHuMIN D continuously released hydrogen peroxide after 12 h to 802 μmol/L and gradually decreased until 24 h. *P. catoniae*, and *S. sanguinis* were aggregated with *L. gasseri* HHuMIN D, and *L. gasseri* HHuMIN D decreased the manufacture of artificial dental plaque from *S. mutans* by 100%. The KB cell adhesion ability of *L. gasseri* HHuMIN D was 4.41 cells per cell and the cell adhesion of *F. nucleatum* and *S. mutans* decreased dramatically in protection and displacement assays. These characteristics of *L. gasseri* HHuMIN D are thought to indirectly help inhibit harmful oral microorganisms. *L. gasseri* HHuMIN D showed direct inhibitory effects on the growth of oral pathogens. *L. gasseri* HHuMIN D does not produce biogenic amines and ammonia, hemolysis and mucin decomposition activity were not observed and antibiotic resistance testing and WGS analysis confirmed that there was no antibiotic resistance or genes associated with biogenic amines, platelet aggregation, virulence or antibiotic resistance. Three genes that express bacteriocin were identified. These assessments demonstrated compliance of the *L. gasseri* HHuMIN D with EFSA and FDA requirements and this study suggests that *L. gasseri* HHuMIN D may be effective in inhibiting harmful oral bacteria and has potential for use as a natural probiotic, a functional food, or a food for wellbeing.

## Data Availability

The datasets used and/or analysed during the current study are available from the corresponding author on reasonable request.

## Abbreviations

*L. gasseri*: *Lactobacillus gasseri*
*F. nucleatum*: *Fusobacterium nucleatum*
*P. gingivalis*: *Porphyromonas gingivalis*
*P. intermedia*: *Prevotella intermedia*
*P. catoniae*: *Porphyromonas catoniae*
*S. sobrinus*: *Streptococcus sobrinus*
*S. mitis*: *Streptococcus mitis*
*S. oralis*: *Streptococcus oralis*
*S. gordonii*: *Streptococcus gordonii*
*S. sanguinis*: *Streptococcus sanguinis*
*S. parasanguinis*: *Streptococcus parasanguinis*
*S. mutans*: *Streptococcus mutans*
WGS: Whole genome sequencing
WHO: World Health Organization
GTase: Glucosyltransferase
VSCs: Volatile sulfur compounds
H_2_S: Hydrogen sulfide
CH_3_SH: Methyl mercaptan
CHX: Chlorhexidine
CPC: Cetylpyridinium chloride
FAO: Food and Agriculture Organization of the United Nations
TMB: Tetra methyl benzidine
DMSO: Dimethyl sulfoxide
MRS: De Man-Rogosa-Sharpe
PBS: Phosphate buffered saline
LBS: *Lactobacillus* Selection
BHI: Brain Heart Infusion
MSB: Mitis Salivarius Sucrose Bacitracin
FBS: Fetal bovine serum
MEM: Minimum critical medium
CFU: Colony-forming unit
CT: Cycle threshold
Trimethoprim: Trimethoprim–sulfamethoxazole
LSM-Cys: LAB susceptibility test medium with L-cysteine
MIC: Minimal inhibitory concentration
COG: Clusters of Orthologous Groups of proteins
KEGG: Kyoto Encyclopedia of Genes and Genomes
CGE: Center for Genomic Epidemiology
CARD: Comprehensive antibiotic resistance database
GTF: Glucosyl transferase
gtf: Glucosyltransferase
GBD: Glucan binding domain
BAs: Biogenic amines
CDSs: Coding sequences
MAT: Methionine adenosyltransferase
